# Supplementary material for: Formulation, inflammation, and RNA sensing impact the immunogenicity of self-amplifying RNA vaccines
Source: Mol Ther Nucleic Acids. 2022 Dec 5;31:29–42. doi: 10.1016/j.omtn.2022.11.024 (PMC9794906; doi:10.1016/j.omtn.2022.11.024)
Supplement: Document S1. Figures S1–S6 [file mmc1.pdf]

OMTN, Volume *31*

Supplemental information

Formulation, inflammation, and RNA sensing impact the immunogenicity of self-amplifying RNA vaccines

John S. Tregoning, David C. Stirling, Ziyin Wang, Katie E. Flight, Jonathan C. Brown, Anna K. Blakney, Paul F. McKay, Robert F. Cunliffe, Valarmathy Murugaiah, Christopher B. Fox, Mitchell Beattie, Ying K. Tam, Cecilia Johansson, and Robin J. Shattock

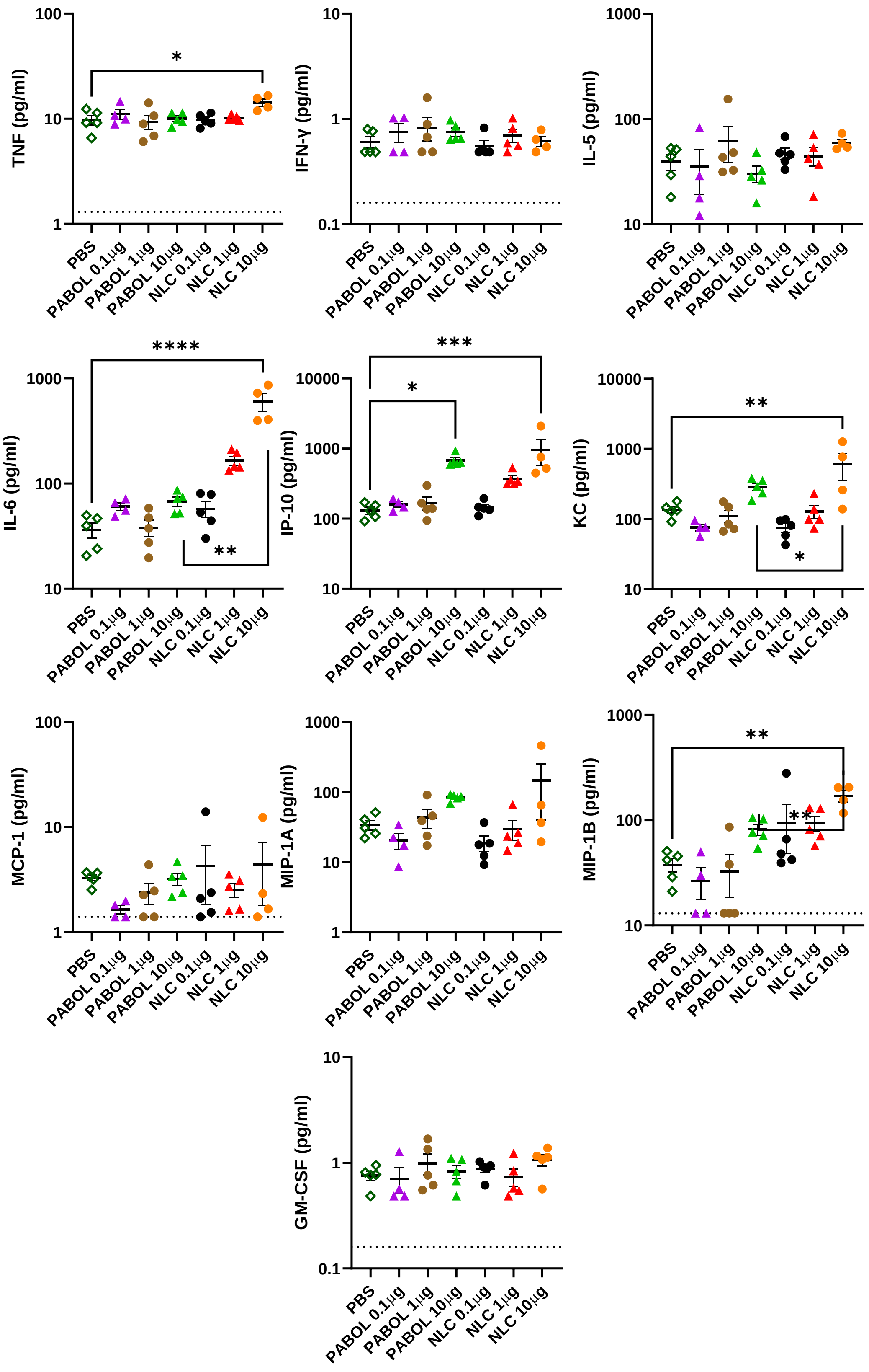

Figure S1. Comparison of blood cytokines after prime immunisation with NLC or pABOL formulated saRNA. Female C57BL/6 mice were intramuscularly immunised with increasing doses of saRNA encoding HA formulated with pABOL or in a nanostructured lipid carrier (NLC) at 0 and 4 weeks. Cytokines in blood were measured by MSD multiplex 4 hours after primary immunisation. Individual cytokine levels. Dotted line where present indicates limit of detection.

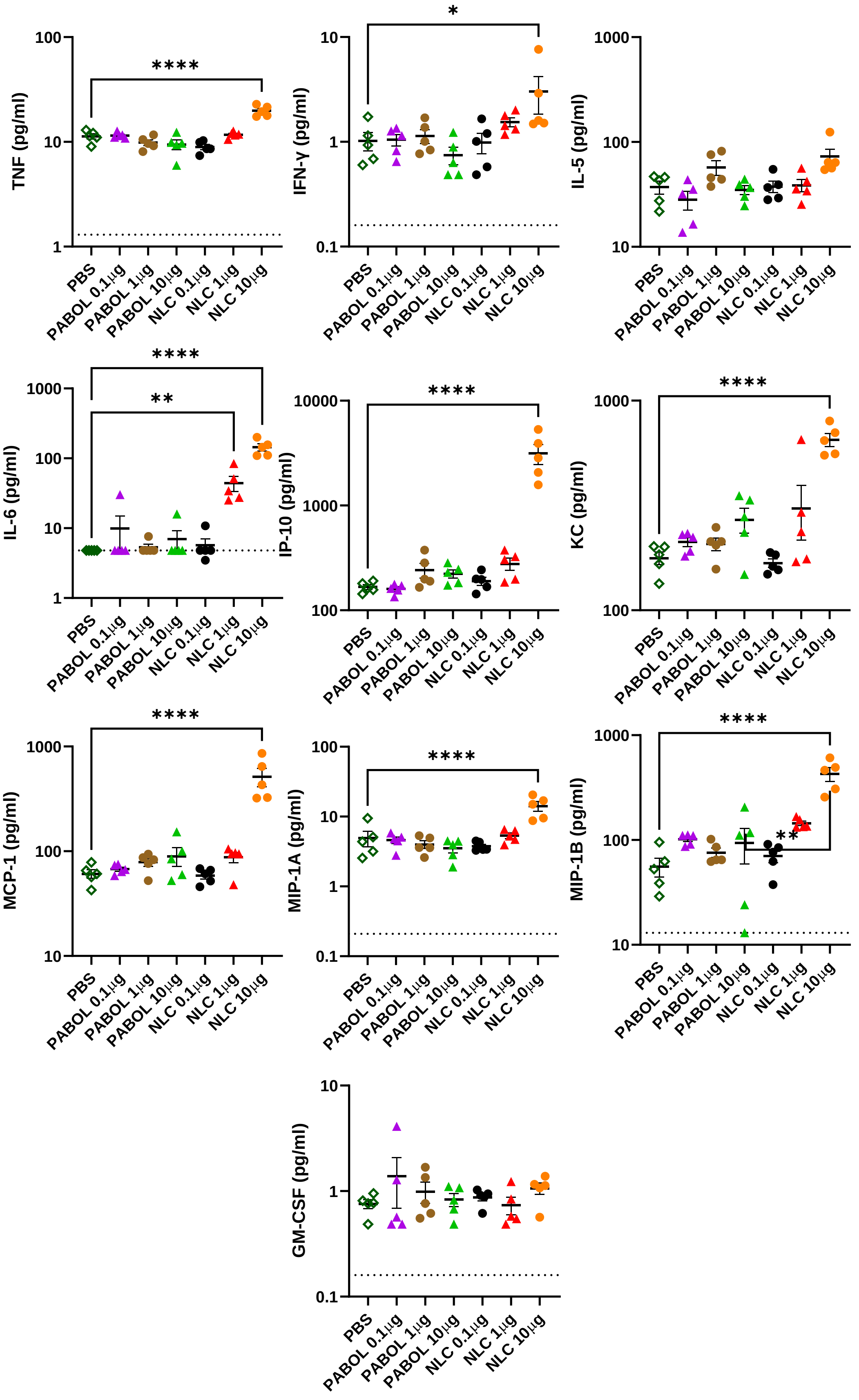

Figure S2. Comparison of blood cytokines after boost immunisation with NLC or pABOL formulated saRNA. Female C57BL/6 mice were intramuscularly immunised with increasing doses of saRNA encoding HA formulated with pABOL or in a nanostructured lipid carrier (NLC) at 0 and 4 weeks. Cytokines in blood were measured by MSD multiplex 4 hours after primary immunisation. Individual cytokine levels.

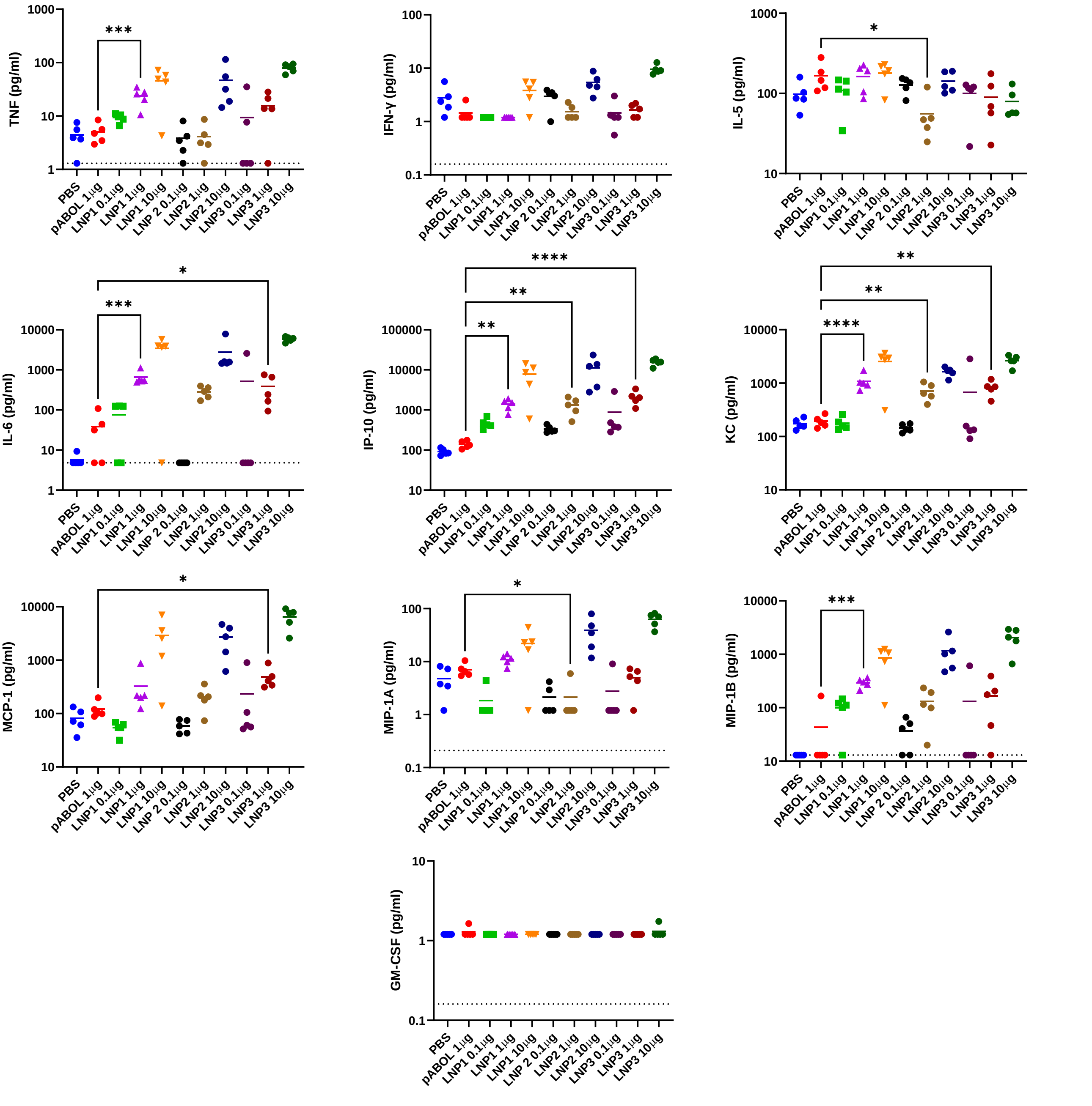

Figure S3. Comparison of blood cytokines after immunisation with LNP or pABOL formulated saRNA. Female C57BL/6 mice were intramuscularly immunised with increasing doses of saRNA encoding HA formulated with lipid nanoparticles (LNP) at 0 and 4 weeks. Cytokines in blood were measured by MSD multiplex 4 hours after primary immunisation. Individual cytokine levels.

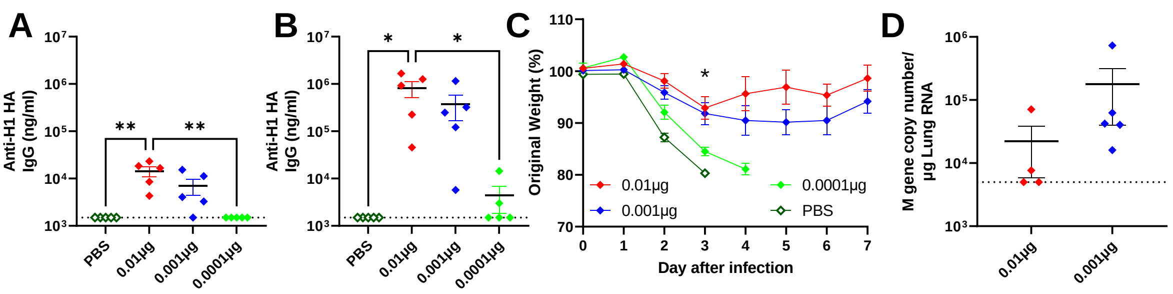

Figure S4. LNP formulated saRNA are protective at a very low dose. Female C57BL/6 mice were intramuscularly immunised with small doses of saRNA encoding HA formulated with lipid nanoparticles (LNP) at 0 and 4 weeks. Blood was collected to measure anti-HA antibody responses at 4 (A) and 6 weeks (B). Mice were infected intranasally with influenza virus at 6 weeks, weight loss was measured after infection (C). Viral load on d7; 0.0001µg and PBS immunised groups culled earlier (D). N=5 mice per group, points represent individual animals (A, B) or means (C), *p<0.05, ** p<0.01. Statistical analysis was performed by ANOVA with a Tukey test.

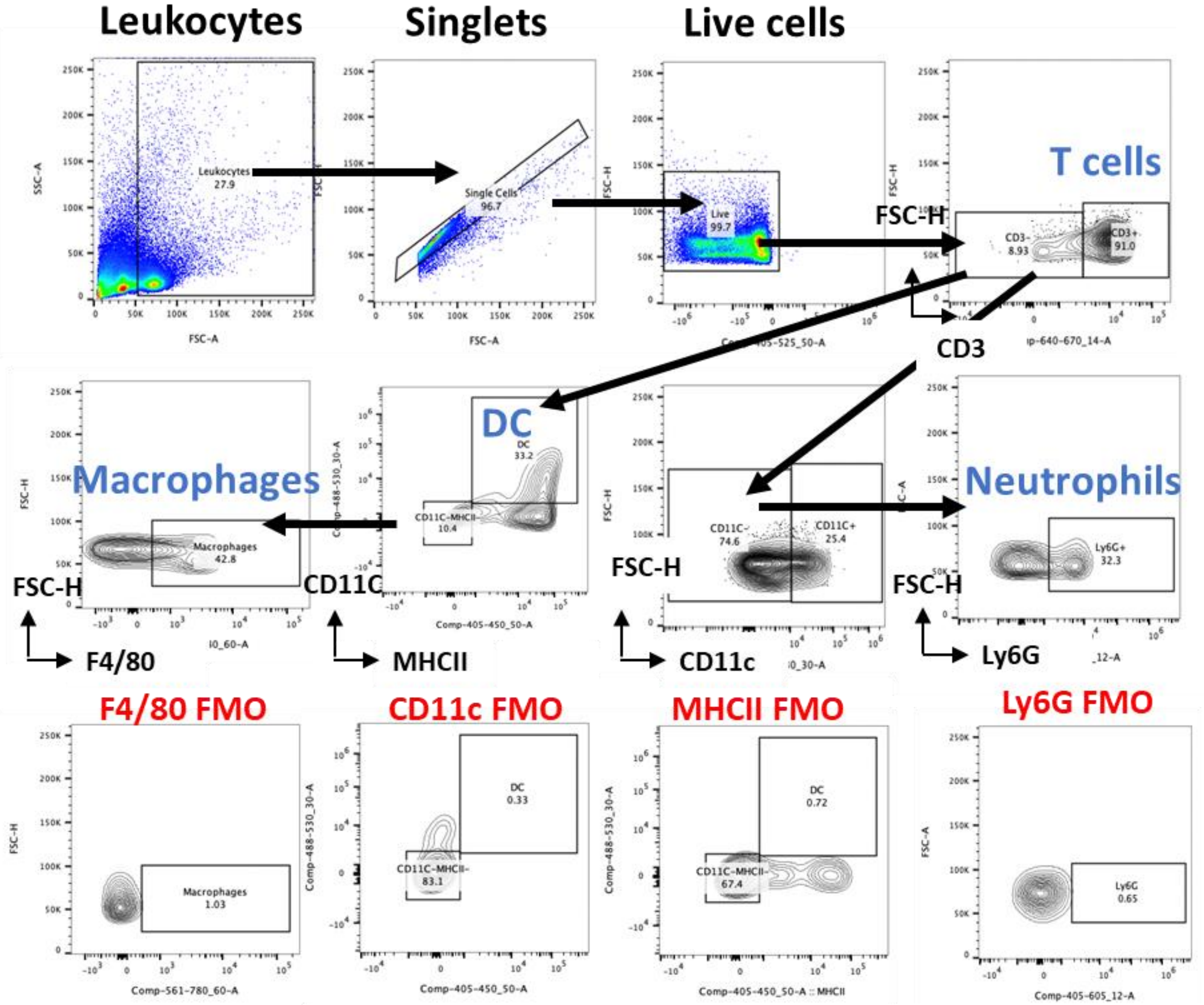

Figure S5. Gating strategy. Cells were extracted from lymph nodes prior to running by flow cytometry. Gating strategy for different cell types shown.

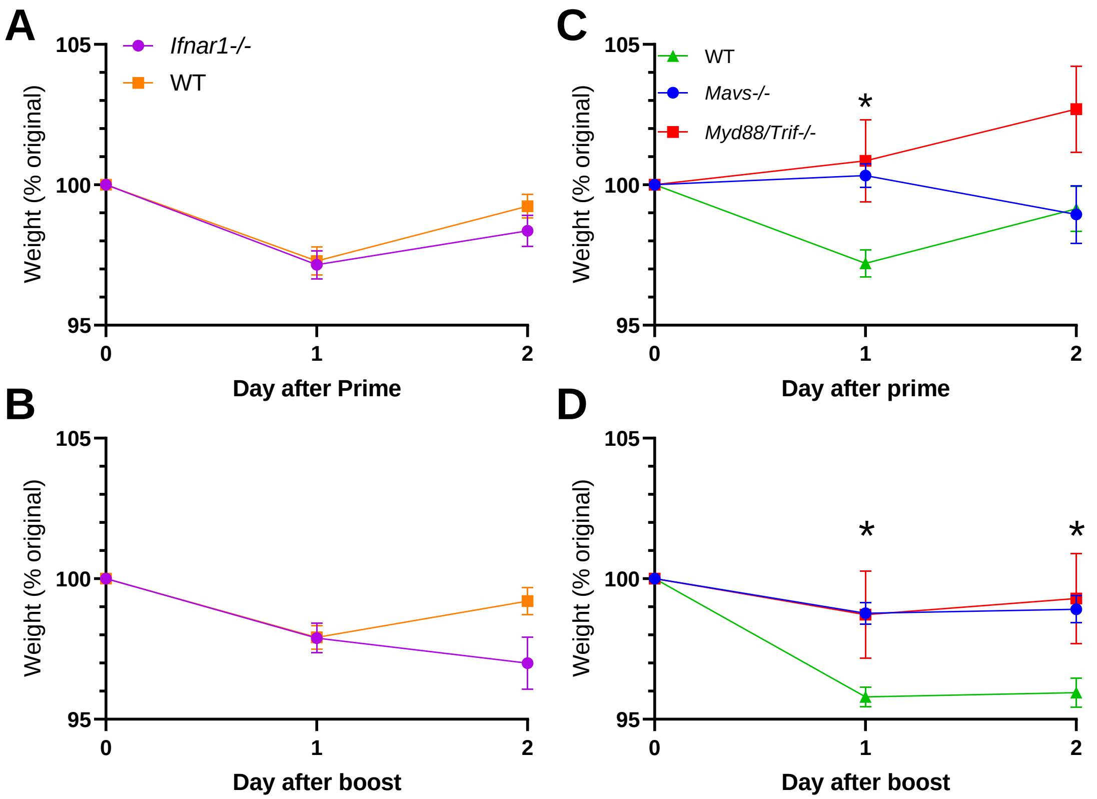

Figure S6. MAVS deficiency affects acute weight loss after vaccination. Wild type and *Ifnar*$^{-/-}$ mice were immunised with 1 µg saRNA formulated in LNP. Weight change was measured after prime (A) or boost immunisation (B). Wild type, *Mavs*$^{-/-}$ and *Myd88/Trif*$^{-/-}$ mice were immunised with 1 µg saRNA formulated in LNP. Weight change was measured after prime (C) or boost immunisation (D). *p<0.05 comparing *Mavs*$^{-/-}$ and wild type.
