# Supplementary material for: Formulation, inflammation, and RNA sensing impact the immunogenicity of self-amplifying RNA vaccines
Source: Mol Ther Nucleic Acids. 2022 Dec 5;31:29–42. doi: 10.1016/j.omtn.2022.11.024 (PMC9794906; doi:10.1016/j.omtn.2022.11.024)
Supplement: Document S2. Article plus supplemental information [file mmc2.pdf]

Molecular Therapy
Nucleic Acids
Original Article

Formulation, inflammation, and RNA sensing impact the immunogenicity of self-amplifying RNA vaccines

John S. Tregoning,[1] David C. Stirling,[1] Ziyin Wang,[1] Katie E. Flight,[1,6] Jonathan C. Brown,[1] Anna K. Blakney,[1,7] Paul F. McKay,[1] Robert F. Cunliffe,[1] Valarmathy Murugaiah,[1] Christopher B. Fox,[2,3] Mitchell Beattie,[4] Ying K. Tam,[4] Cecilia Johansson,[5] and Robin J. Shattock[1]

[1]Department of Infectious Disease, Imperial College London, St. Mary's Campus, London, UK; [2]IDRI, Seattle, WA, USA; [3]Department of Global Health, University of Washington, Seattle, WA, USA; [4]Acuitas Therapeutics, 6190 Agronomy Road, Ste 405, Vancouver, BC, Canada; [5]National Heart and Lung Institute, Imperial College London, St. Mary's Campus, London, UK

To be effective, RNA vaccines require both *in situ* translation and the induction of an immune response to recruit cells to the site of immunization. These factors can pull in opposite directions with the inflammation reducing expression of the vaccine antigen. We investigated how formulation affects the acute systemic cytokine response to a self-amplifying RNA (saRNA) vaccine. We compared a cationic polymer (pABOL), a lipid emulsion (nanostructured lipid carrier, NLC), and three lipid nanoparticles (LNP). After immunization, we measured serum cytokines and compared the response to induced antibodies against influenza virus. Formulations that induced a greater cytokine response induced a greater antibody response, with a significant correlation between IP-10, MCP-1, KC, and antigen-specific antibody titers. We then investigated how innate immune sensing and signaling impacted the adaptive immune response to vaccination with LNP-formulated saRNA. Mice that lacked MAVS and are unable to signal through RIG-I-like receptors had an altered cytokine response to saRNA vaccination and had significantly greater antibody responses than wild-type mice. This indicates that the inflammation induced by formulated saRNA vaccines is not solely deleterious in the induction of antibody responses and that targeting specific aspects of RNA vaccine sensing might improve the quality of the response.

INTRODUCTION

The COVID-19 pandemic has highlighted the potential of RNA vaccines as a flexible platform that can be rapidly deployed to control emerging infections.[1] The first experimental SARS-CoV-2 mRNA vaccine was administered 63 days after the publication of the viral sequence. [2] An alternative to mRNA vaccines is self-amplifying RNA (saRNA)-based vaccines. saRNA are based on alphaviruses, with a replicase complex that amplifies both the transgene and the vaccine construct. A major benefit of the saRNA platform compared with non-replicating mRNA is a reduction in the dose needed to induce a protective immune response. We have seen a significant dose-reduction in pre-clinical models where protection was induced with 100-fold less saRNA than mRNA.[3] Reduced dose translates to more vaccines per production run, accelerating rollout, which is particularly important during a pandemic. However, moving from mouse models[4] into clinical trials[5] had mixed results with 13% of vaccinated individuals not responding. Understanding why saRNA vaccines do not always lead to adaptive immunity is key to the effective deployment of this extremely promising platform.

One factor that could influence the immune response to RNA vaccines is the balance between expression of the vaccine-encoded antigen and inflammation induced by the vaccine. RNA vaccines differ from conventional protein vaccines because they need to be translated *in situ* before the antigen can be detected by the adaptive immune system. If the RNA is not translated, there can be no immune response to the vaccine.[6] This balance between inflammation and expression is an inherent feature of RNA vaccines because they are made of foreign RNA, which is sensed by pattern recognition receptors (PRRs) including those from the Toll-like receptor (TLR) and RIG-I-like receptor (RLR) families.[7] The downstream response to activation of these receptors is complex,[8] but two key downstream processes are the induction of the type I interferon response and the activation of the inflammatory NF-κB pathway. The inflammation induced by RNA vaccines can be beneficial in terms of recruitment and activation of antigen-presenting cells and cells from the adaptive immune system, but detrimental when it inhibits translation. Boosting the recruitment and activation of the downstream immune response while limiting the negative impact on antigen expression is a key goal in the development of better RNA vaccines.[9] One way to

Received 14 February 2022; accepted 30 November 2022;
https://doi.org/10.1016/j.omtn.2022.11.024.

[6]Present address: UCL, UK
[7]Present address: UBC, Canada
Correspondence: John S. Tregoning, Department of Infectious Disease, Imperial College London, St. Mary's Campus, London, UK.
E-mail: john.tregoning@imperial.ac.uk

This is an open access article under the CC BY license (http://creativecommons.org/licenses/by/4.0/).

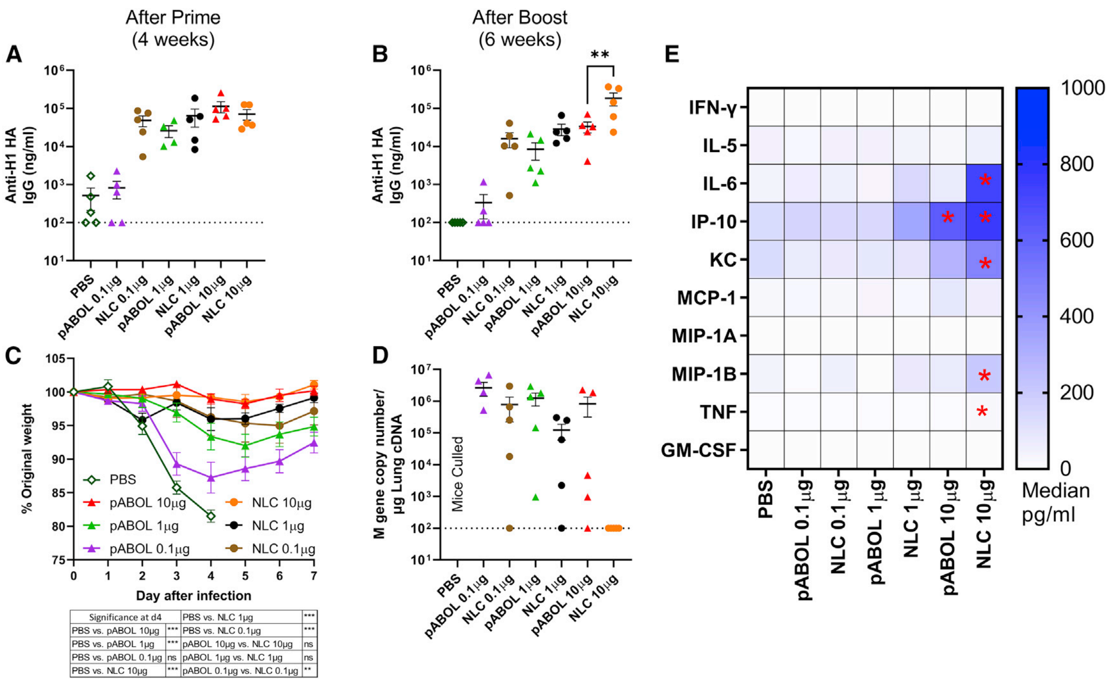

Figure 1. NLC-formulated saRNA induces a better protective response than pABOL, associated with increased cytokine responses

(A–E) Female C57BL/6 mice were intramuscularly immunized with increasing doses of saRNA encoding HA formulated with pABOL or in a nanostructured lipid carrier (NLC) at 0 and 4 weeks. Blood was collected to measure anti-HA antibody responses at 4 (A) and 6 weeks (B). Mice were infected intranasally with influenza virus at 6 weeks, and weight loss was measured after infection (C) and viral load at day 7 after infection (D). Cytokines in blood were measured by MSD multiplex 4 h after primary immunization. Cells represent medians: in cell *$p < 0.05$ vs. PBS (E). N = 5 mice per group, where points represent individual animals (A, B, D) or means (C), **$p < 0.01$, ***$p < 0.001$; dotted line represents limit of detection. Error bars represent SEM. Study performed once. Statistical analysis was performed by ANOVA with a Tukey test.

understand this response is to measure and correlate the systemic cytokine response immediately after immunization as a way to predict the magnitude of the adaptive immune response.[10]

Understanding which pathways are beneficial and which are inhibitory may then help in the rational development of new strategies. One area that might influence the response is the formulation of the RNA vaccine. RNA as a charged hydrophilic molecule needs some form of assistance to get into cells; this is particularly important with saRNA constructs that are much longer because of the incorporation of the alphavirus replication machinery. As with DNA vaccines, cellular uptake can be achieved by electroporation, but more commonly, cell entry is enabled via formulation. Two broad formulation approaches can be adopted: mixing with a cationic polymer to neutralize the charge or incorporation into a lipid particle to enable transition of the cell membrane.[11] Lipids, particularly lipid nanoparticles, have been widely used, including in the COVID-19 mRNA vaccines from Pfizer/BioNTech (COMIRNATY) and Moderna (Spikevax). The interaction of formulation, RNA, and innate immunity are going to be important variables to be optimized.

In the current study, we compared three different formulations for saRNA, a cationic polymer (pABOL[12]), a lipid emulsion (nanostructured lipid carrier, NLC[13]), and three lipid nanoparticles (LNPs). We investigated the early inflammatory profile after immunization and explored how that related to downstream adaptive immune responses. We then explored the role of RNA sensing on the adaptive immune response to vaccination. We observed that LNP gave the strongest immune response and that signaling of the LNP/saRNA construct through RLR pathways may be detrimental to the downstream adaptive immune response.

RESULTS

saRNA formulated with the polymer pABOL induces a protective immune response

We wanted to investigate the relationship between vaccine-induced cytokines in the blood and the adaptive immune response to the encoded antigen. We have previously demonstrated that when saRNA is formulated with the bio-reducible polymer pABOL, it can induce a systemic cytokine response and protect against influenza virus infection.[14] We compared responses to an NLC that has been shown to provide protection against Zika virus. C57BL/6 mice were immunized intramuscularly with increasing amounts (0.1 µg, 1.0 µg, and 10 µg) of saRNA formulated with pABOL or NLC in a prime-boost regime at 0 and 4 weeks, before intranasal challenge with H1N1 influenza virus at 6 weeks. Sera was collected after 4 and 6 weeks, and anti-HA IgG responses were assessed by ELISA. There was a dose response in antibody titers following vaccination with

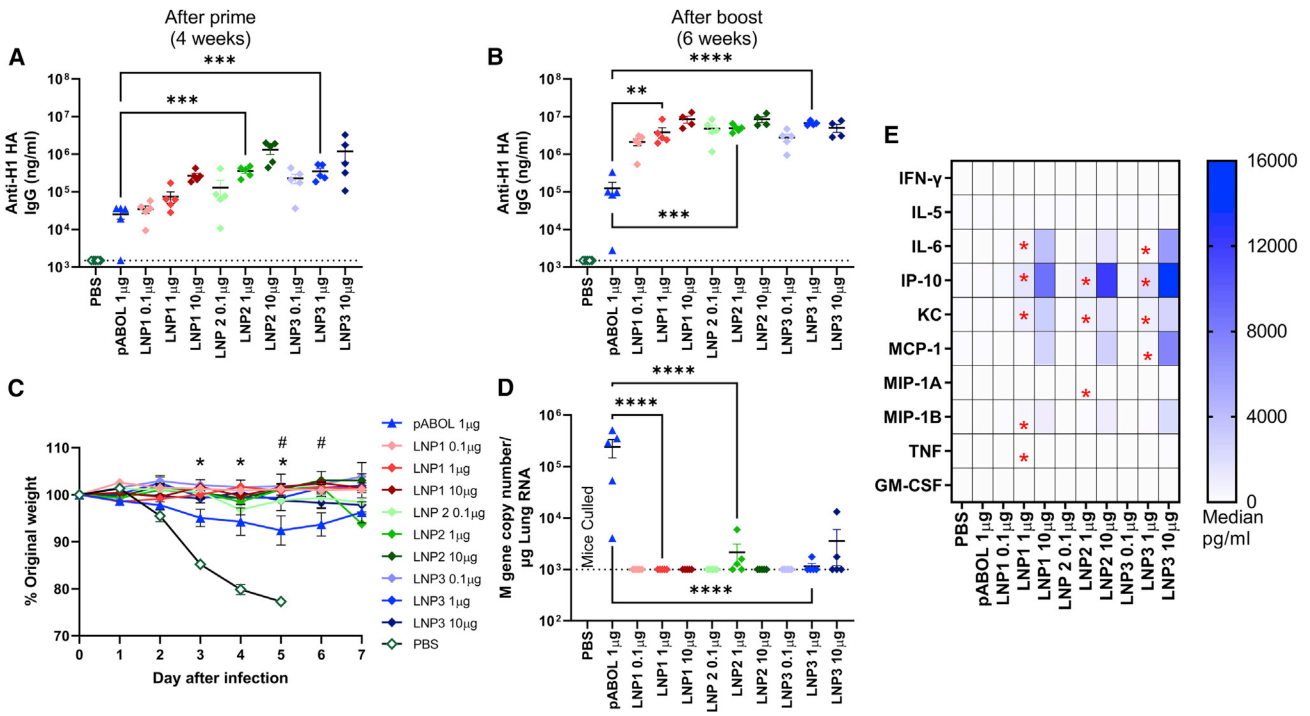

Figure 2. LNP-formulated saRNA induces a better protective response than pABOL, associated with increased cytokine responses

(A–D) Female C57BL/6 mice were intramuscularly immunized with increasing doses of saRNA encoding HA formulated with lipid nanoparticles (LNPs) at 0 and 4 weeks. Blood was collected to measure anti-HA antibody responses at 4 (A) and 6 weeks (B). Mice were infected intranasally with influenza virus at 6 weeks, and weight loss was measured after infection (C) and viral load at day 7 after infection (D). Cytokines in blood were measured by MSD multiplex 4 h after primary immunization. Cells represent medians: in cell *$p < 0.05$ 1 µg LNP vs. 1 µg pABOL (E). N = 5 mice per group, where points represent individual animals (A, B, and D) or means (C). (A, B, and D) **$p < 0.01$, ***$p < 0.001$, ****$p < 0.0001$ as indicated; (C) *$p < 0.05$ between PBS and vaccine groups; #$p < 0.05$ between 1 µg LNP and pABOL groups; error bars represent SEM. Study performed once. Statistical analysis was performed by ANOVA with a Tukey test.

pABOL-formulated saRNA, with 10 µg inducing significantly more antigen-specific antibodies than control mice after the prime ($p < 0.01$, Figure 1A). In general, NLC antibody responses were greater than the equivalent dose of pABOL-formulated RNA, with a significant increase in the 10-µg dose compared with pABOL after the boost ($p < 0.01$, Figure 1B).

Mice were infected with 3×10^4 pfu A/California/7/2009 (H1N1) influenza virus; following infection, we measured weight change as a readout of disease severity.[15] Mice immunized with 10 µg pABOL saRNA did not lose any weight after infection. Both the 10 µg and 1 µg groups, but not the 0.1 µg group, lost significantly less weight than the control group at day 4 after infection, at which point the control group was culled because they had reached allowed severity ($p < 0.001$, Figure 1C). All three of the NLC groups were protected against disease, losing significantly less weight than the PBS group at day 4 after infection ($p < 0.001$). Interestingly protection was induced with a much lower dose of NLC-formulated saRNA than pABOL, with the 0.1 µg group losing significantly less weight on day 4 after infection ($p < 0.01$). It is of note that all groups of mice receiving pABOL-formulated saRNA had detectable influenza virus RNA in the lungs on day 7 after infection (Figure 1D). The high-dose NLC group had cleared the virus, and there was a reduction in detectable virus in the 1 µg NLC group.

We investigated the acute inflammatory cytokine profile in the blood at 4 h after immunization. As with the antibody, there was a dose response in the cytokines detected in the blood after immunization (Figures 1E and S1). 10 µg pABOL induced a significantly greater level of IP-10 after the prime immunization than PBS, and 10 µg NLC induced significantly greater levels of IL-6, IP-10, KC, MIP-1B, and TNF than PBS. The level of IL-6, KC, MIP-1B, and TNF in the blood was significantly greater after 10 µg saRNA in NLC than the administration of 10 µg saRNA in pABOL. A similar pattern of cytokine response was seen after the booster vaccination (Figure S2) These results suggest that formulations that can induce a systemic cytokine inflammation can boost the antibody response to saRNA vaccines.

saRNA in an LNP induces more inflammation than pABOL

Having observed that there was an association between the cytokines induced by saRNA vaccine formulations and the downstream antibody response, we explored the association with another formulation platform that has been widely used for clinical RNA vaccine

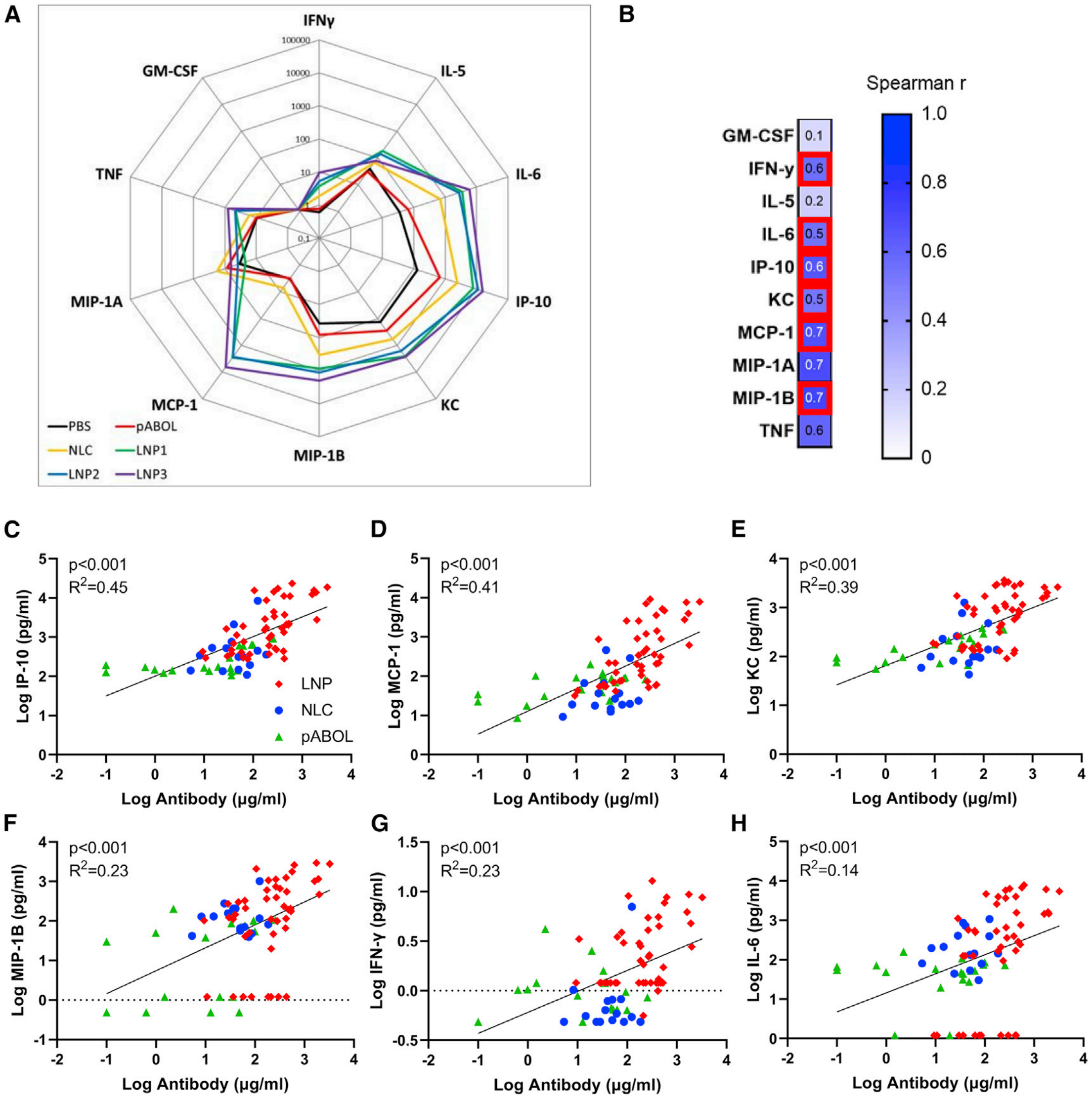

Figure 3. Systemic antibody responses correlate closely with level of cytokine induced after immunization

(A–G) Pooled analysis of data from studies presented in Figures 1 and 2: cytokine assessed by MSD in blood collected 4 h after immunization with saRNA in different formulations. Spider diagram comparing 10 μg groups, where each point represents mean (A). Comparison of antibody and cytokine response, where data are presented by formulation, pABOL in green, NLC in blue, and LNP in red, but correlation on all data (B). Correlations for IP-10 (C), KC (D), MCP-1 (E), MIP-1B (F), IFN-γ (G), and IL-6 (H). Combined data from two studies, n = 90 animals in total. Simple linear regression (B–H).

delivery: LNPs. C57BL/6 mice were immunized intramuscularly with increasing amounts (0.1 μg, 1.0 μg, and 10 μg) of saRNA formulated with three different LNP in a prime-boost regime at 0 and 4 weeks, before intranasal challenge with H1N1 influenza virus at 6 weeks. Sera was collected after 4 and 6 weeks, and anti-HA responses were assessed by ELISA. There was a dose response in antigen-specific antibody levels following vaccination with LNP1- and LNP2-formulated saRNA, with the 10-μg dose inducing a significantly higher antibody response than the lower doses after a single immunization (Figure 2A). We used 1 μg pABOL-formulated saRNA as a comparator, where both 1 μg LNP1 and LNP3 led to a significantly higher antibody response than pABOL-formulated vaccine. Booster vaccination increased the antibody response, with all three LNP formulations leading to a significantly greater antibody response than the equivalent dose of pABOL-formulated saRNA (Figure 2B). 6 weeks after the first immunization, the mice were challenged with intranasal influenza virus. All doses of all three LNP formulations led to significant protection against disease following infection ($p < 0.05$, days 3–5; Figure 2C). Mice immunized with 1 μg LNP-formulated RNA lost significantly less weight on days 5 and 6 after infection than the pABOL-immunized mice. While the pABOL immunized mice still had detectable viral RNA after infection, most of the LNP-immunized

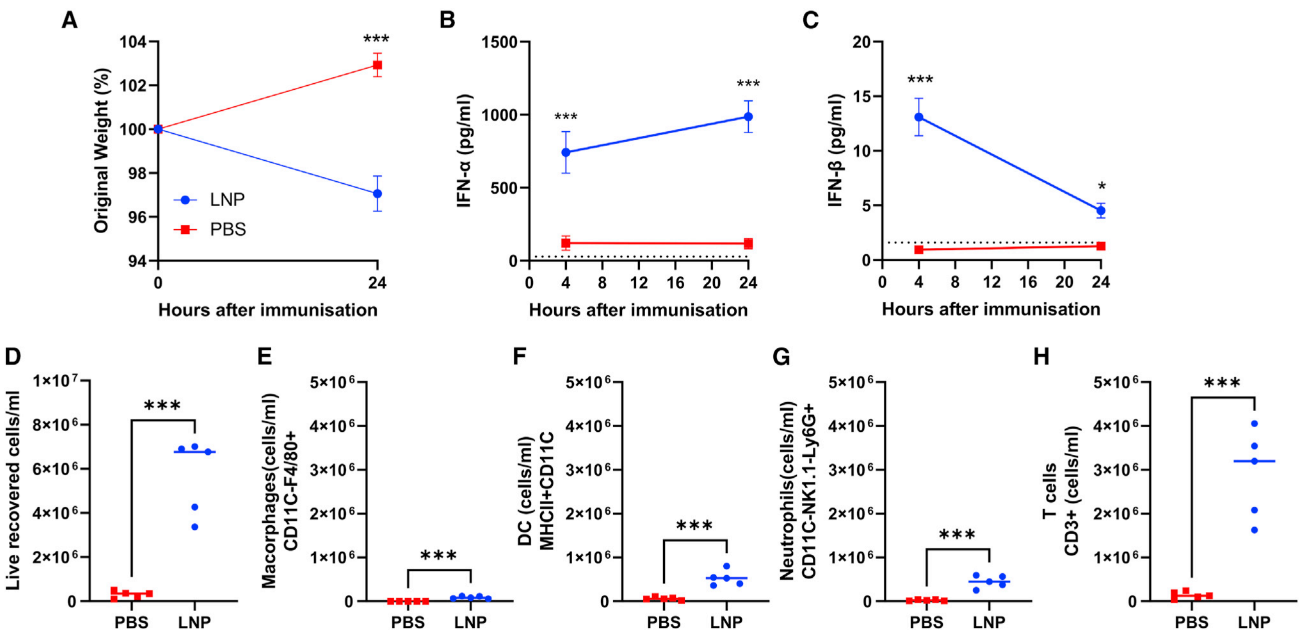

Figure 4. LNP-formulated RNA induces a type I IFN response and significant cell recruitment to the lymph nodes 24 h after immunization

(A–H) Female C57BL/6 mice were intramuscularly immunized with 1 μg saRNA encoding HA formulated in LNP. Mice were weighed after immunization (A). IFN-α (B) and IFN-β (C) measured in blood at 4 and 24 h after immunization. Cells were collected from lymph nodes 24 h after immunization and analyzed by flow cytometry, with total cell count (D), macrophages (E), cDC1 (F), neutrophils (G), and T cells (H). N = 5 mice per group, where points represent individual animals (D–H) or means (A–C). $^{**}p < 0.01$, $^{***}p < 0.001$, $^{****}p < 0.0001$ as indicated; error bars represent SEM. Study performed once. Statistical analysis by two way ANOVA (A–C) and t test (D–H).

mice had no detectable influenza virus RNA on day 7 after infection (Figure 2D). Blood was taken 4 h after primary immunization to determine the impact of the formulation on the systemic cytokine response. There was a dose response in the cytokines detected in the blood after immunization (Figures 2E and S2). LNP-formulated saRNA induced IL-6, IP-10, KC, MCP-1, and MIP-1B in a dose-responsive manner. The 10-μg dose of each LNP induced significantly more IFN-γ, IL-6, IP-10, KC, MCP1, MIP-1A, MIP-1B, and TNF in the blood than PBS. Comparing the 1-μg dose with 1 μg pABOL, all three LNPs induced significantly greater levels of KC and IP-10 (Figure S3).

While the two studies were performed separately, comparing the overall pattern of responses (Figure 3), there was approximately a log-fold greater level of cytokine detected in the blood to LNPs than either NLC or pABOL. The cytokines IL-6, IP-10, and KC predominated the response to all three formulations (Figure 3A), with MCP-1 also at higher levels in LNP-immunized animals. We explored whether there was a correlation between cytokines and antibody response (Figure 3B). All data from animals described above were grouped from the three different formulations and PBS treatment. For all nine of the cytokines measured in the blood, there was a significant correlation with the amount of antibody measured at week 4 after immunization. The strongest correlation was with IP-10 (Figure 3C), MCP-1 (Figure 3D), and KC (Figure 3E); there was a weak correlation with MIP-1B (Figure 3F), IFN-γ (Figure 3G), and IL-6 (Figure 3H). A pattern of higher antibody and cytokines could be seen with LNPs > NLC > pABOL.

We focused on the LNPs for further investigation, as these showed a significantly greater response. To assess whether the inflammation induced by vaccination had a systemic effect, we measured weight after immunization, and we saw a slight but significant weight loss 24 h after immunization, suggesting systemic inflammation and reactogenicity (Figure 4A). IFN-α increased from 4 to 24 h after immunization and was significantly greater than the control group (Figure 4B). IFN-β peaked at 4 h after immunization and was significantly greater than the control group (Figure 4C). We then investigated the infiltration of immune cells to the draining lymph node 24 h after immunization (gating strategy; Figure S5). There was a significant increase in cell numbers recovered (Figure 4D), with significant increases in macrophages (Figure 4E), dendritic cells (DCs) (Figure 4F), neutrophils (Figure 4G), and T cells (Figure 4H). The inflammation following immunization with LNP-formulated saRNA therefore induces an acute recruitment of cells to the lymph nodes.

One potential advantage of saRNA is in dose sparing. We compared protection against influenza virus infection with small amounts of LNP-formulated saRNA using 0.01, 0.001, or 0.0001 μg saRNA formulated in LNP3. There was a dose response in anti-HA IgG at both 4 (Figure S4A) and 6 (Figure S4B)

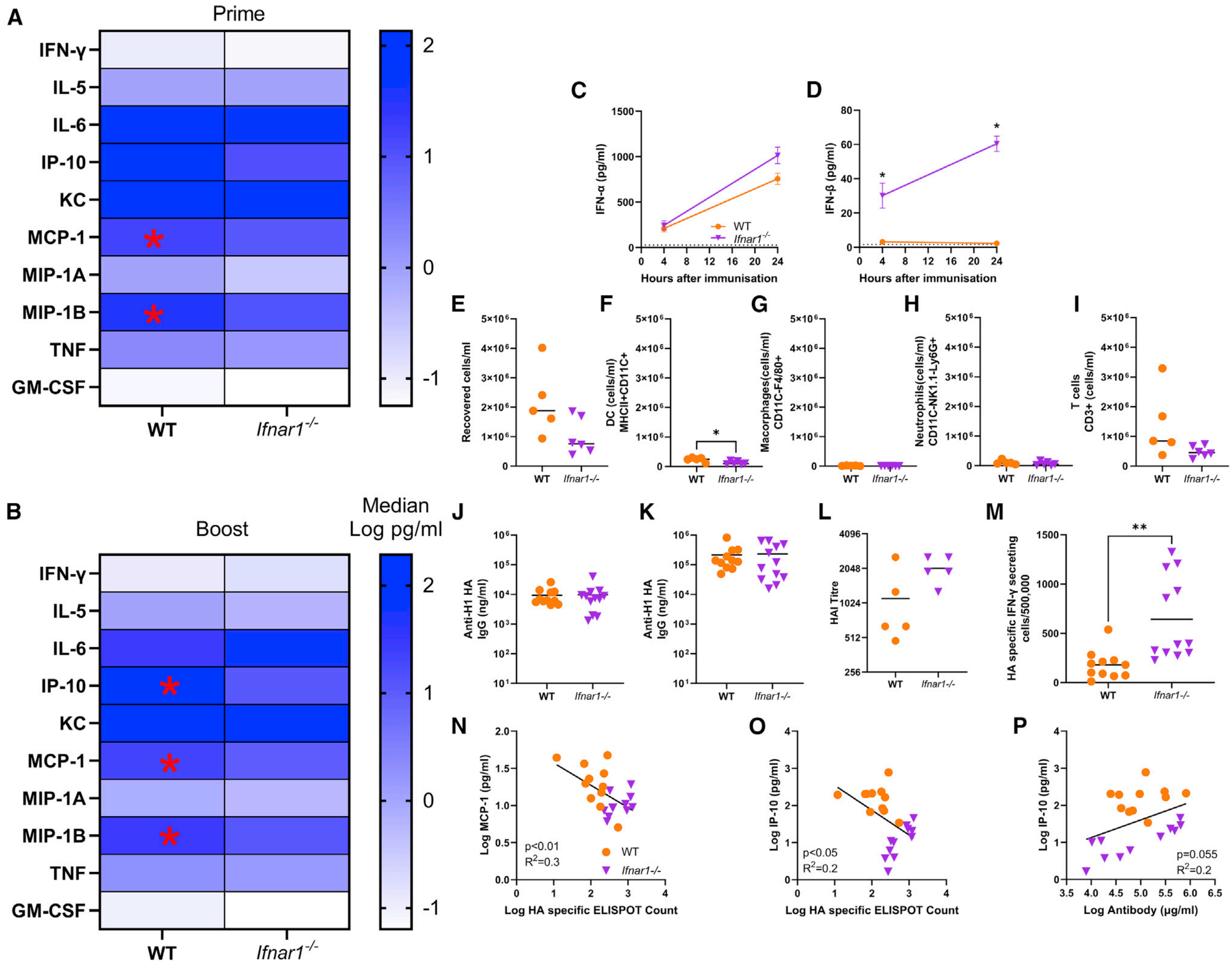

Figure 5. Mice lacking IFN-α receptor signaling have altered cytokine responses and increased T cell response to saRNA vaccination

(A and B) Wild-type and *Ifnar1*$^{-/-}$ mice were immunized with 1 μg saRNA formulated in LNPs. 4 h after prime (A) and boost (B), cytokines were measured in blood. Cells represent medians: in cell *$p < 0.05$ WT vs. *Ifnar1*$^{-/-}$. (C–I) FN-α (C) and IFN-β (D) 4 and 24 h after primary immunization. Cells were collected from lymph nodes 24 h after immunization and analyzed by flow cytometry, with total cell count (E), macrophages (F), cDC1 (G), neutrophils (H), and T cells (I). (J–P) Blood was collected for analysis of HA-specific antibody 4 (J) and 6 (K) weeks after start of study and HAI at 6 weeks (L); 6 weeks after the study, spleens were collected and assessed for HA-specific T cells by ELISPOT (M). Blood cytokines at 4 h after boost immunization were compared with T cell response for MCP-1 (N) and IP-10 (O) and antibody against IP-10 (P). Data from two experiments combined, N ≥ 5 mice per group, where points represent individual animals, *$p < 0.05$, **$p < 0.01$, ***$p < 0.001$; error bars represent SEM. Statistical analysis was performed by ANOVA with Tukey test (A–D) and unpaired t-test (E–M); correlation on log-transformed data was by simple linear regression.

weeks after first immunization. At 6 weeks after the first immunization, mice were infected intranasally with H1N1 influenza virus. While all animals in the PBS control group had to be culled at day 3 and all animals in the 0.0001 μg group had to be culled on day 4, animals immunized with either 0.01 or 0.001 μg saRNA were protected against severe disease. Both the 0.01 and 0.001 μg groups lost significantly less weight than the naive control or 0.0001 μg groups on day 3 after infection ($p < 0.05$, Figure S4C), but all animals in both the naive and 0.0001 μg groups had to be culled by day 4 because of excess weight loss. There was some influenza viral RNA detected in the lungs of the animals in the 0.01 and 0.001 μg groups on day 7 after infection (Figure S4D).

Impact of type I interferon signaling on response to saRNA vaccine

Having seen that different formulations affect the inflammatory response to saRNA, we investigated how early events in sensing and responding to saRNA shape the immune response. Since saRNA are foreign RNA, they are likely to induce a type I IFN response that may affect the expression of the encoded antigen, as well as the recruitment and activation of immune cells to the site of

immunization. We therefore compared the response in mice lacking the type I interferon receptor 1 (IFNAR1) through which IFN-α and IFN-β signal. Responses were compared with wild-type (WT) mice of the same background (C57BL/6), and mice were immunized with 1 μg saRNA formulated in LNP 3. There was a mild, transient weight loss 1 day after prime and boost immunization, with no difference between the two strains (Figures S6A and S6B). Blood was collected 4 h after both the prime and the boost immunization. Immunization induced a range of cytokines in both WT and *Ifnar1*$^{-/-}$ mice. At prime, WT mice had significantly more of the chemokines MCP-1 and MIP-1B in the blood (Figure 5A). 4 h after booster immunization a similar pattern was seen (Figure 5B) with significantly greater levels of MCP-1, MIP-1B, and IP-10. There was no difference in levels of IFN-α between WT and *Ifnar1*$^{-/-}$ mice (Figure 5C); interestingly there was significantly more IFN-β in *Ifnar1*$^{-/-}$ mice at both 4 and 24 h after prime immunization (Figure 5D). We compared the impact of type I IFN signaling on the cellular recruitment to the draining lymph node. There was no difference in the number of cells recovered from lymph nodes (Figure 5E). *Ifnar1*$^{-/-}$ mice had significantly fewer DCs (Figure 5F), but no differences in macrophages (Figure 5G), neutrophils (Figure 5H), or T cells (Figure 5G).

Adaptive immune responses were assessed by ELISA and ELISPOT at 6 weeks after first immunization; infectious challenge was not undertaken because the underlying gene knockout in *Ifnar1*$^{-/-}$ mice would have affected susceptibility to infection independent of vaccine responses. [16] There was no significant difference in antibody responses between the two strains of mice either at 4 weeks (single dose) (Figure 5J) or 6 weeks (two doses) (Figure 5K) after immunization. Neither was there any change in hemagglutination inhibition (HAI) titer (Figure 5L). However, the *Ifnar1*$^{-/-}$ had significantly more HA-specific IFN-γ secreting T cells isolated from the spleen measured by ELISPOT (Figure 5M). In post-hoc analysis, we compared the T cell response to the cytokines that were significantly different between the two strains. There was a significant inverse correlation between the level of MCP-1 (Figure 5N) and IP-10 (Figure 5O) and the IFN-γ producing T cell count. There was also a significant correlation between IP-10 and antibody (Figure 5P).

Impact of saRNA vaccine sensing on adaptive immune response

We then investigated whether the way in which saRNA was sensed impacted the outcome. Mice lacking the RLR adaptor MAVS or a combined knockout for the TLR adaptors MyD88 and TRIF (*Myd88/Trif*$^{-/-}$) were compared with WT littermate mice. Mice were immunized with 1 μg saRNA formulated in LNP 3. There was a mild, transient weight loss in WT mice 1 day after prime and boost immunization, but not in the *Mavs*$^{-/-}$ or *Myd88/Trif*$^{-/-}$ mice (Figures S6C and S6D), suggesting that the inflammation was associated with reduced systemic effects. Blood was collected 4 h after both the prime and the boost immunization. Immunization induced a range of cytokines in both WT and knockout mice. At prime, there was a trend in the *Myd88/Trif*$^{-/-}$ animals toward reduced levels of IL-6 and TNF, although the differences were not statistically significant (Figure 6A). 4 h after booster immunization, there were significant differences in the responses between the three strains (Figure 6B). The *Mavs*$^{-/-}$ mice had significantly reduced IL-5 than WT mice. WT mice had significantly more IL-6 and KC than *Myd88/Trif*$^{-/-}$ mice and more KC than *Mavs*$^{-/-}$ mice. *Mavs*$^{-/-}$ mice had significantly less IFN-α at 4 and 24 h after primary immunization than WT mice, but there was no difference between WT and *Myd88/Trif*$^{-/-}$ mice in IFN-α. There was no difference in IFN-β (Figure 6D). After primary immunization there was no difference in IFN-γ between the strains (Figure 6E), but after boost immunization, there was significantly more IFN-γ in *Mavs*$^{-/-}$ mice (Figure 6F). 24 h after primary immunization, we observed a significantly greater number of cells from the lymph nodes of *Mavs*$^{-/-}$ mice (Figure 6G). There were significantly more T cells (Figure 6H) and DCs (Figure 6I), but no difference in macrophages (Figure 6J) or neutrophils (Figure 6I).

Adaptive immune responses were assessed at 4 and 6 weeks after first immunization. As with the *Ifnar1*$^{-/-}$ mice, it was not possible to perform a challenge because of the underlying gene knockouts. [17,18] *Mavs*$^{-/-}$ mice had significantly greater antibody responses after prime immunization than either *MyD88/Trif*$^{-/-}$ or WT mice ($p < 0.05$, Figure 7A). After a booster vaccine, a similar effect was seen, and the antibody response was significantly greater in *Mavs*$^{-/-}$ mice than *Myd88/Trif*$^{-/-}$ or WT mice (Figure 7B). There was also significantly more HAI in *Mavs*$^{-/-}$ mice than WT (Figure 7C). There were significantly more HA specific IFN-γ producing cells from the spleens of *Mavs*$^{-/-}$ mice than *Myd88/Trif*$^{-/-}$ (Figure 7D). We performed post-hoc analysis of the cytokines that were significantly different after booster immunization with the antibody and T cell responses. IFN-γ, IL-6, and KC (Figures 7E–7G) correlated significantly with antibody titer, and IFN-γ and IL-6 but not KC correlated significantly with the T cell response (Figures 7H–7J).

DISCUSSION

In this study we investigated the interplay of RNA vaccine-induced inflammation and the downstream adaptive immune response. We observed that the formulations that increased systemic cytokine responses at the time of immunization led to increased antibody responses and protection against influenza virus infection. The level of cytokines in the blood correlated with the antigen-specific antibody responses. We subsequently investigated the interface of innate immune sensing of the RNA vaccine and the downstream adaptive immune response. Mice lacking the IFNAR1 receptor that binds IFN-alpha and beta had an altered response, with significantly less MCP-1, MIP-1B, and IP-10 but significantly greater specific T cell responses. Interestingly mice lacking the MAVS adaptor, which is important for the RLR sensing pathway, had significantly increased IFN-γ and decreased IL-5 and KC responses after immunization with increased antibody responses after the primary immunization. These data suggest that the sensing of formulated saRNA vaccine material by the innate immune system is critical in inducing the adaptive immune response and that some inflammatory responses are beneficial, but others can be limiting.

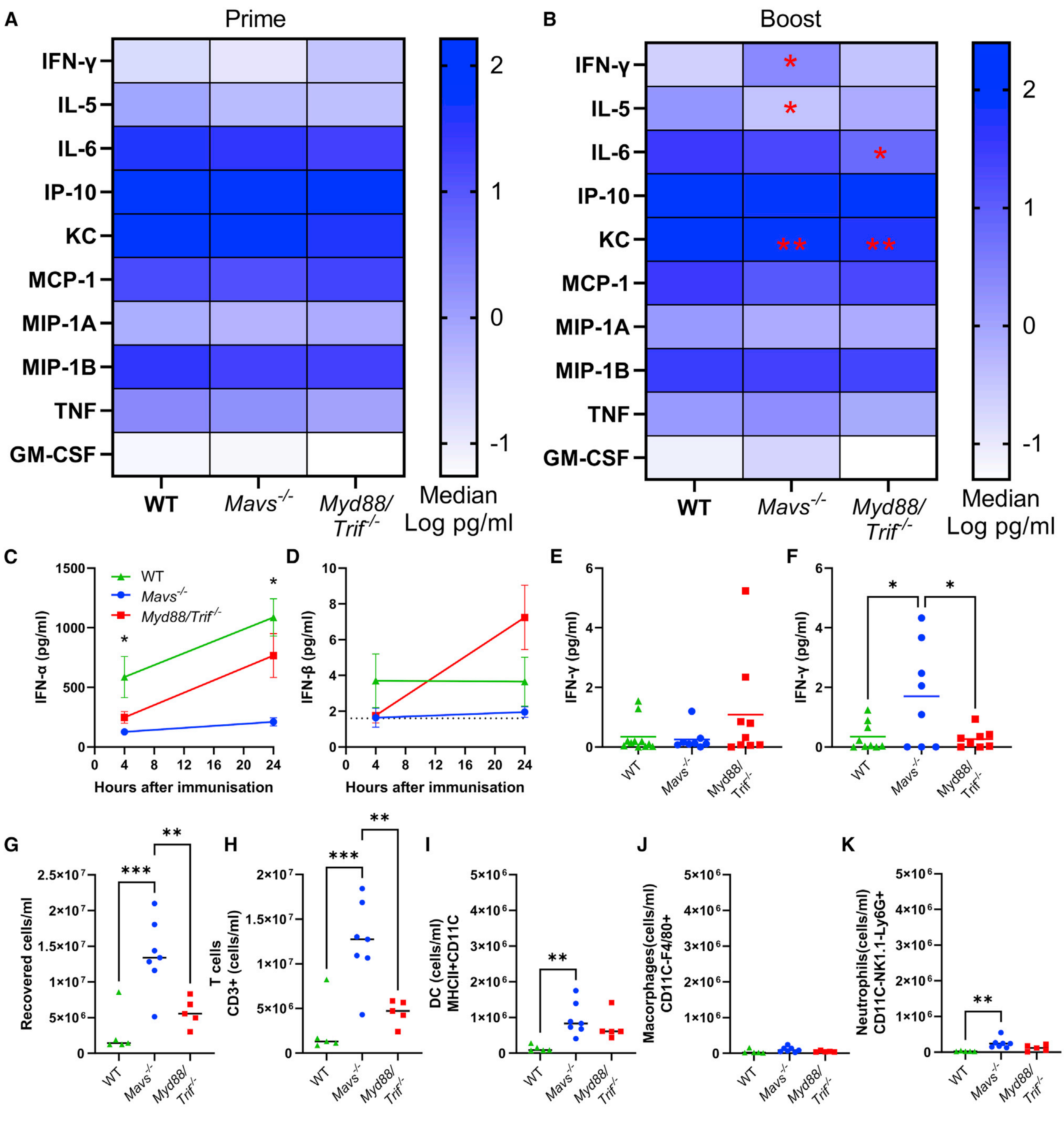

Figure 6. Mice lacking MAVS or MyD88/TRIF have altered innate responses to saRNA vaccination

(A–D) Wild-type, *Mavs*$^{-/-}$, and *Myd88/Trif*$^{-/-}$ mice were immunized with 1 μg saRNA formulated in LNPs. 4 h after prime (A) and boost (B), cytokines were measured in blood. Cells represent medians: in cell *$p < 0.05$ vs. WT. IFN-α (C) and IFN-β (D) 4 and 24 h after primary immunization. IFN-γ 4 h after prime (E) and boost (F). Cells were collected from lymph nodes (G–K) 24 h after immunization and analyzed by flow cytometry, with total cell count (G), T cells (H), DCs (I), macrophages (J), and neutrophils (K). Data from two experiments combined (A, B, E, and F) and data from a single study (C, D, and G–K), N ≥ 5 mice per group, where points represent individual animals, *$p < 0.05$, **$p < 0.01$, ***$p < 0.001$; error bars represent SEM. Statistical analysis was performed by ANOVA with Tukey test; correlation on log-transformed data was by simple linear regression.

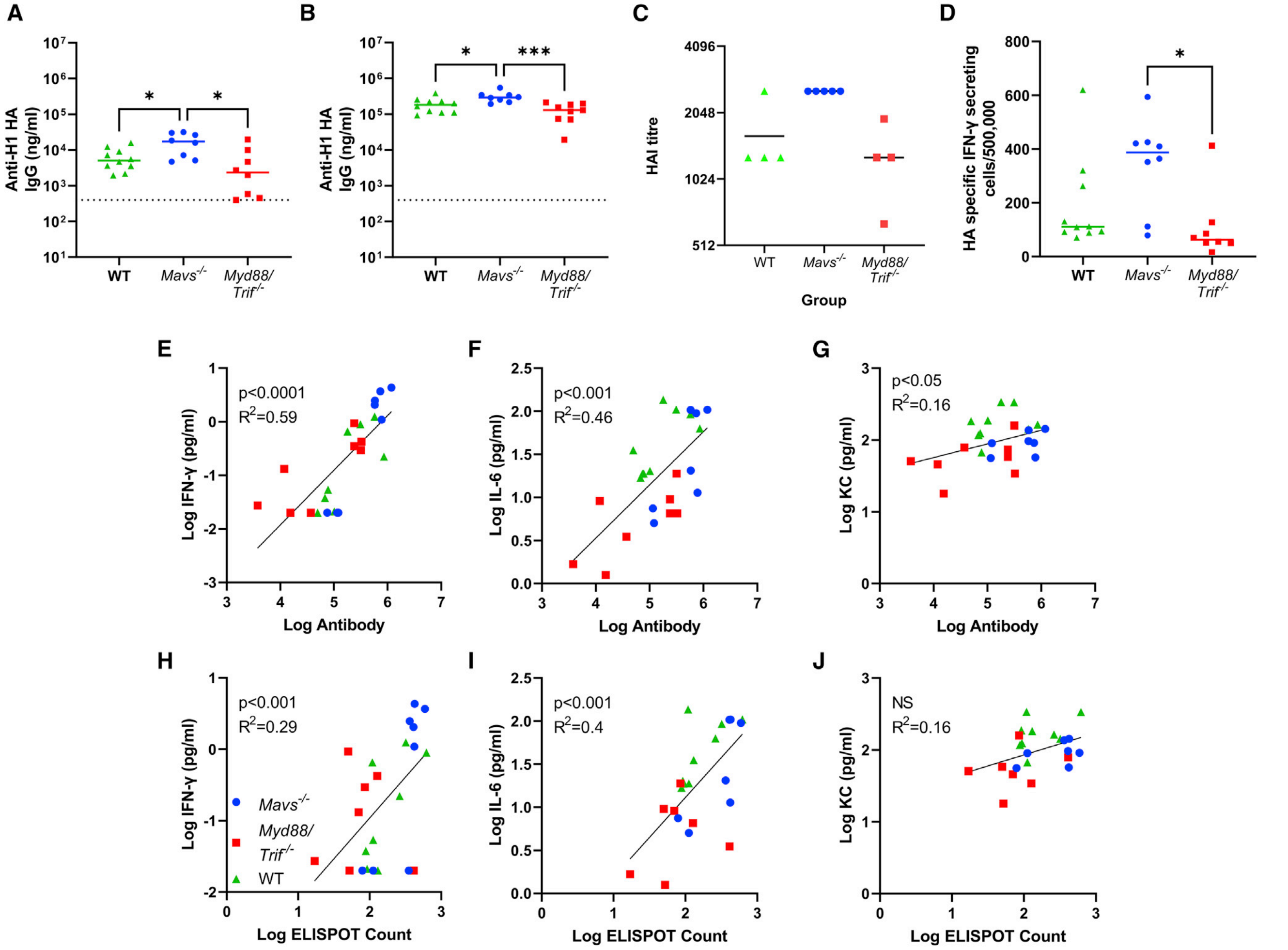

Figure 7. Mice lacking MAVS have altered adaptive responses to saRNA vaccination

(A–D) Wild-type, *Mavs*$^{-/-}$, and *Myd88/Trif*$^{-/-}$ mice were immunized with 1 μg saRNA formulated in LNP. Blood was collected for analysis of HA-specific antibody 4 (A) and 6 (B) weeks after start of study; HAI assessed 6 weeks after the start of the study (C); 6 weeks after the study spleens were collected and assessed for HA-specific T cells by ELISPOT (D). (E–J) Blood cytokine at 4 h after boost immunization was compared with antibody at boost for IFN-γ (E), IL-6 (F), and KC (G) and T cells responses for IFN-γ (H), IL-6 (I), and KC (J). Data from two experiments combined, where points represent individual animals, *$p < 0.05$, **$p < 0.01$, ***$p < 0.001$. Statistical analysis was performed by ANOVA with Tukey test; correlation on log-transformed data was by simple linear regression.

In this and other studies, we have tested a range of different saRNA vaccine formulations. In these studies, the most potent formulation of the saRNA vaccine was with LNPs. LNPs were used in the first in human clinical study using an saRNA[5] as well as in the licensed mRNA vaccines from Pfizer/BioNTech and Moderna.[2] In our study, we observed that the LNP-formulated saRNA induced higher levels of inflammation than polymer-formulated saRNA. Other studies have also observed systemic inflammation following administration of RNA formulated in LNP in mice[14,19] and NHP.[20] Understanding what drives this inflammation is important in designing the next generation of RNA vaccines. An important question is whether the formulation itself or the way in which it delivers the RNA shapes the immune response to RNA vaccines. There is some suggestion that the LNP themselves are inflammatory, which given their similarity to oil-in-water-type formulations and the inflammatory nature of nanoparticles is conceptually possible. Intramuscular immunization with LNP alone induced upregulation of a number of inflammatory genes in the muscle.[21] In a preprint, injection of LNPs led to some cellular recruitment, though the study did not perform a detailed characterization of empty LNPs.[22] However, in another *in vitro* study, empty LNPs were not inflammatory when combined with isolated mouse DCs.[23] We did not directly explore the role of the physicochemical characteristics of the different formulations on inflammation, but these have been previously shown to have a significant impact on the delivery of protein antigens and as such should be expected to have a similar impact on RNA vaccines,[24] as has changing the ionizable lipid component of the LNPs.[25] LNPs were originally designed as a method for silent delivery of RNA for therapeutic

approaches, but the data presented here and elsewhere suggests that inflammation is important for their role in vaccines, which may relate to the specific formulation of the LNP and their properties. The clinically successful vaccines may have hit a sweet spot between being immunogenic and able to deliver cargo, though one observation from the clinical studies is a relatively short-lived antibody response; whether this is a feature of RNA vaccines or formulation is yet to be seen.

The role of the formulation compared with the RNA cargo in the induction of inflammation is an important consideration. Foreign RNA is in and of itself inflammatory, triggering both inflammatory cytokines and the type I/III IFN responses,[7] so inflammation may reflect the efficiency of RNA cargo delivery. The destination of the encapsulated RNA may be critical in the downstream response; LNPs deliver RNA through both phagocytosis and pinocytosis and potentially fusion with the cell membrane. We observed different patterns of both cytokine induction and adaptive immunity in the TLR and RLR adaptor knockout mice, suggesting both pathways are involved in the response, which may indicate that the RNA is in both the cytosol and the endosome, but further research is required to confirm this. A study comparing saRNA delivered by LNPs or electroporation saw a very different kinetic both in terms of the transgene expression and the IFN-β response, with longer transgene expression after electroporation, which may correlate with significantly lower IFN-β at the injection site.[26] It is not clear what is driving this difference: it might be associated with where the RNA is in the cell. It was of interest that there was significantly more IFN-β in the blood of *Ifnar1*$^{-/-}$ than control mice; this may reflect increased replication of the saRNA construct or a failure to regulate the IFN response, where a similar increase in sera IFN has been seen in West Nile virus infection.[27] Whether differences seen between the different formulations were a product of improved RNA delivery, different inflammatory profiles, or altered cellular location of delivery is not clear.

To investigate the role of sensing of the LNP-formulated saRNA vaccine, we looked in gene knockout mice. Initially we looked at *Ifnar1*$^{-/-}$ mice, through which receptor type I IFNs signal. These animals had reduced IP-10, MCP-1, and MIP-1B and increased T cell responses. A similar decrease in IP-10 was seen using a different LNP containing an saRNA expressing the RSV-F gene,[21] but in our system, there was no impact of type I IFN signaling on antibody responses. Why type I IFN signaling only reduces T cell responses and not antibody production in our model needs further investigation. We then wanted to investigate how the sensing of the RNA/LNPs affected the immune response. Different patterns were seen in the different knockout mice, and in mice lacking the TLR adaptors MyD88 and TRIF, there was significantly less IL-6 and KC induction after immunization. A recent study demonstrated cytokine release following LNP exposure of bone marrow-derived DCs was TLR4 mediated,[28] though this was with a different LNP formulation. In mice lacking the RLR adaptor MAVS, there was significantly more IFN-γ and reduced IL-5, with a significant increase in antibody to the vaccine. Interestingly in another study looking at gene expression using the SEAP reporter gene following LNP delivery, there was no difference in either *Tlr7*$^{-/-}$ or *Mavs*$^{-/-}$ knockout mice, so potentially the effect is not on gene expression but on the downstream immune response.[21] The basis for the difference seen between these two different pattern recognition pathways is not immediately clear; both induce NF-κB, IRF3, and IRF7. Since TLRs additionally trigger IRF5 and AP-1, these may drive the cytokines that are beneficial for the recruitment of immune cells.[7] Interestingly, incorporation of viral genes that specifically target the MDA5 pathway (ORF4a from MERS and V from PIV-5) increase saRNA transgene expression *in vitro* and *in vivo* as well as increasing immunogenicity,[29] while other proteins targeting other aspects of the RNA sensing machinery had no effect. The studies performed here focused on the LNP-formulated saRNA, so responses and the impact of innate signaling need to be considered in the context of the whole complex, not just the RNA component. As described above, differences may be driven by where and how the LNP/RNA complex enters the cells and may not be about the downstream signaling from the different receptors; more research is required to understand the differences seen. Another area for further investigation is the role of individual cytokines in the context of both WT and PRR knockout mice, as different patterns of response were seen; whether the cytokines have a functional role or a bystander marker of something else needs further investigation. IP-10 has been associated with better antibody responses following human RNA vaccination, as part of a signature with IL-15 and IFN-γ.[30]

An interesting finding was that the cytokine profile in response to the LNP-formulated vaccines was similar to that induced by TLR ligands *in vitro* human cells,[31] in mice[32] and NHP.[33] We saw a similar response to an MF-59-formulated influenza vaccine[10] with similar correlations between MCP-1 and KC and the antibody response. However, these cytokines may be biomarkers of a global inflammatory response to vaccination and not necessary for the induction of an adaptive response. As we observed in the current study, decreased MCP-1 in the *Ifnar1*$^{-/-}$ mice was associated with increased T cell responses. What is interesting is that in the mouse model, greater cytokine responses in the blood to LNP-formulated RNA vaccine were associated with better adaptive immune response, and pABOL-formulated RNA induced fewer systemic cytokines and a poorer antibody response. A recent study using LNP-formulated mRNA vaccine demonstrated that vaccine-induced IL-6 plays a role in the induction of T follicular helper cells and germinal center B cells.[25] We also saw a significant correlation between IFN-γ and antibody response, with elevated IFN-γ in *Mavs*$^{-/-}$ knockout mice after secondary immunization; a similar effect has been seen following immunization with an LNP-formulated pseudouridine-containing mRNA vaccine.[34] Interestingly, there was a difference in the downstream T cell response in the published data from MAVS knockout mice immunized with a silenced mRNA vaccine, and in our data using saRNA, we observed increased T cell responses by ELISPOT, where the mRNA study saw increased IFN-γ-producing CD8+ T cells by flow cytometry; this may reflect the differences in the two vaccine types or methodology of readout. Because of the need for translation of the RNA cargo, the received wisdom is that inflammation may not be beneficial,[9] and a

number of strategies have been proposed to alter the response,[9] including the incorporation of steroids within the LNP.[35] But these approaches may be too broad acting and act to inhibit the beneficial cytokines in addition to those that dampen expression, though different strategies will be needed for therapeutic proteins than RNA vaccines. We saw a modest but significant acute weight loss following vaccination in WT mice that was not observed in either the RLR or TLR signaling knockout mice, suggesting the inflammation has some systemic effects.

One of the limitations that affect interpretation of these data in the context of translation into clinical practice is the difference between innate sensing of RNA in mice and humans. For example, TLR8 in inbred mice has a different expression pattern and sensitivity to ligands than human TLR8,[36] lacking five amino acids in the ectodomain that may contribute to functionality.[37] In a recent study, we observed that ferrets have a variable response to saRNA vaccines, and they have a more similar TLR8 to humans, which supports the idea that sensing is important.[38] So, the balance between inflammation and vaccine expression may differ. Another limitation of the study is that we were unable to investigate protection against challenge in the knockout mice used because of the underlying differences in response to the virus itself.

The COVID-19 pandemic accelerated a wave of vaccine innovation,[2] with a number of platforms that had previously only been in pre-clinical and early clinical studies rapidly moving into efficacy trials and deployment. Of these platforms, RNA vaccines were the most rapid to be deployed and among the most effective. However, it is still a relatively new technology, and improvements can be made for both manufacturing and immunogenicity. In this study, we investigated the role of LNP/saRNA sensing and vaccine-induced inflammation on the adaptive immune response. We observed that the induction of systemic cytokines after immunization was associated with a greater antibody immune response and protection against infection. Dissecting the innate response through the use of gene knockout mice suggested that vaccine-induced inflammation is required for an adaptive immune response. Understanding how this applies to humans is the critical next step in further optimizing RNA vaccines, where formulations that get the balance between recruitment of immune cells and suppression of vaccine expression will be crucial.

MATERIALS AND METHODS

saRNA construct

saRNA was synthesized from a backbone plasmid vector based on a Trinidad donkey Venezuelan equine encephalitis strain (VEEV) alphavirus genome as previously described.[4] The gene of interest for *in vivo* immunogenicity studies was hemagglutinin from the H1N1 A/California/07/2009 strain.

In vitro transcription of saRNA

Briefly, uncapped RNA was prepared using 1 μg of linearized DNA template in a MEGAScript reaction (Ambion, UK) according to the manufacturer's protocol. Transcripts were then purified by overnight LiCl precipitation at −20°C, pelleted by centrifugation at 14,000 RPM for 20 min at 4°C, washed once with 70% ethanol, centrifuged at 14,000 RPM for 5 min at 4°C, and then resuspended in UltraPure H_2O (Ambion, UK). Purified transcripts were then capped using the ScriptCap m7G capping system (CellScript, Madison, WI, USA) and ScriptCap 2′-0-methyltransferase kit (CellScript) simultaneously according to the manufacturer's protocol. Capped transcripts were then purified by LiCl precipitation, as detailed above, resuspended in UltraPure H_2O, and stored at −80°C until formulation.

Vaccine formulation

pABOL (M_w = 8 kDa) polyplexes were prepared by the "titration method" as previously described.[12] Briefly, in a typical preparation, 5 μL of a stock solution of saRNA (1 mg/mL) was diluted in 35 μL of HEPES buffer (20 mM HEPES, 5 wt % glucose in water, pH 7.4). A volume of 4.5 μL of pABOL stock solution was diluted in 5.5 μL of HEPES buffer. The saRNA solution was then added to the polymer solution (mixed at 1,200 RPM) at a rate of 160 μL/min, for a final N:P ratio of 45:1. Previous studies have shown these particles are ∼200 nm in diameter with a zeta potential of 5 mV.[12]

The NLC formulation was prepared as described previously.[13] Briefly, an oil phase consisting of squalene, glyceryl trimyristate, sorbitan monostearate, and DOTAP (N-[1-(2,3-dioleoyloxy)propyl]-N,N,N-trimethylammonium chloride) was heated and sonicated at 60°C, and it was combined with a heated aqueous phase consisting of polysorbate 80 in citrate buffer using high-shear mixing. The formulation was subsequently processed by high-pressure homogenization and terminally filtered through a 0.2-μm membrane. Immediately prior to immunization, the NLC was complexed with the aqueous RNA at an N:P ratio of 15:1. Previous studies have shown these NLC particles to be ∼40 nm in diameter with a zeta potential of 28 mV.[13]

Lipid nanoparticle production was achieved by rapid mixing of an ethanolic solution of 1,2-distearoyl-sn-glycero-3-phosphocholine, cholesterol, a PEG-lipid, and an ionizable cationic lipid with saRNA dissolved in an acidic citrate or acetate buffer using an in-line mixer. The PEG and LNP compositions are described in patent application WO 2015/199952; the ionizable lipids for LNP 1, 2, and 3 are described in patent applications WO 2015/199952, WO 2017/004143, and WO 2017/075531, respectively. The post-in-line formulation was subjected to Tangential Flow Filtration (Repligen, Waltham WA) to remove the ethanol and exchange to 1x PBS. The formulation was then filtered through a 0.2-micron filter and frozen at −80°C in the presence of a cryoprotectant. The LNP had an average hydrodynamic diameter of ∼85 nm with a monodisperse distribution with a PDI <0.1 measured by dynamic light scattering (Malvern NanoZS Zetasizer, Malvern, UK); the LNPs were neutral at physiological pH. The encapsulation efficiency was >90% when measured by the Ribogreen assay (Life Technologies, Carlsbad, CA).

Mouse immunization and infection

For WT, 6- to 8-week-old female C57BL/6 mice were obtained from Charles River (Stirling, UK) and kept in specific-pathogen-free conditions in accordance with United Kingdom's Home Office guidelines. All work was approved by the Animal Welfare and Ethical Review Board (AWERB) at Imperial College, London.

For gene knockout mice, mice deficient in IFNAR1 (*Ifnar1*$^{-/-}$) were obtained from Caetano Reis e Sousa (Crick Institute, UK).[39] Mice deficient in MAVS (Mavs$^{-/-}$) or deficient in MyD88/TRIF (*Myd88/Trif*$^{-/-}$) and control mice obtained from S. Akira (World Premier International Immunology Frontier Research Center, Osaka University, Osaka, Japan)[40] were bred and maintained in specific pathogen-free conditions. Both strains were Ifna6 gfp/+, but since Ifna6 expression was not a primary readout, the mice are designated as WT, Mavs$^{-/-}$, and *Myd88/Trif*$^{-/-}$ mice. The mice were gender and age matched (7–12 weeks) in each experiment.

Mice were injected with 50 μL intramuscular formulated saRNA or PBS as a control. Blood was collected 4 h after immunization for cytokine analysis.

For infections, mice were anesthetized using isoflurane followed by intranasal application with 3×10^4 pfu A/California/7/2009 (H1N1) influenza virus from Prof. Wendy Barclay. Virus was grown in MDCK cells, in serum-free DMEM supplemented with 1 μg/mL trypsin. Mice were culled for humane reasons if they lost more than 80% weight.

Tissue and cell recovery and isolation

Lymph nodes were removed and digested in DNase (200 μg/mL) for 15 min before being homogenized by passage through 100-μm cell strainers, then centrifuged at 500 × *g* for 5 min. Supernatants were removed, and the cell pellet was treated with red blood cell lysis buffer (ACK; 0.15 M ammonium chloride, 1 M potassium hydrogen carbonate, and 0.01 mM EDTA, pH 7.2) before centrifugation at 200 × *g* for 5 min. The remaining cells were resuspended in RPMI 1640 medium with 10% fetal calf serum, and viable cell numbers were determined by trypan blue exclusion.

Flow cytometry

Live lymph node cells were plated out onto a U-shaped 96-well plate then spanned down at 2,000 rpm for 2 min at 4°C. 100 μL of Live/Dead violet dye (BioLegend, Catalog: 423113) was added for 20 min at 4°C in the dark, then centrifuged at 2,000 rpm for 2 min, and the supernatant was taken off. The cell pellet was resuspended in Fc block (Anti-CD16, BD, catalog: 6266549) in PBS-1% BSA for 20 min and stained with the following surface antibodies Ly6G (7046845 BD), CD11c (6209870, BD), F4/80 (15-4801-82, eBiosciences), CD103 (BioLegend, catalog: 121406), NK1.1 (BD Biosciences, catalog: 551114), and MHCII (4289686, eBiosciences) for 1 hour in the dark at room temperature. The excess antibodies were washed off with 1% BSA in PBS three times before being filtered through the fluorescence-activated cell sorting tubes. This was performed on an LSR Fortessa Flow cytometer (BD) and FlowJo. Fluorescent minor one controls were used for surface stains. Analysis was performed using FlowJo.

Multiplex cytokine measurements

Cytokines in blood were assessed using a custom mouse kit from Meso Scale Discovery (MSD) as a 10-spot U-PLEX kit (K15069L-2), including the following analytes (lower limit of detection in pg/mL in parenthesis): GM-CSF (0.16), IFN-γ (0.16), IL-5 (0.63), IL-6 (4.8), IP-10 (0.5), KC (0.43), MCP-1 (1.4), MIP-1α (0.21), MIP-1β (13), and TNF (1.3). IFNα (140) and IFNβ (1.6) were measured using a custom MSD 2 spot U-PLEX kit (K15069L-1).

Semi-quantitative antigen-specific ELISA

Antibodies specific to influenza H1N1 virus were measured in sera using a standardized ELISA. MaxiSorp 96-well plates (Nunc) were coated with 1 μg/mL H1N1 surface protein or a combination of anti-murine lambda and kappa light chain specific antibodies (AbD Serotec, Oxford, UK) and incubated overnight at 4°C. Plates were blocked with 1% BSA in PBS. Bound IgG was detected using A61horseradish peroxidase-conjugated goat anti-mouse IgG (AbD Serotec). Alternatively, IgG1 or IgG2a were detected using subtype-specific secondary antibodies. A dilution series of recombinant murine immunoglobulin was used as a standard to quantify specific antibodies. 3,3′,5,5′-Tetramethylbenzidine (TMB) with H_2SO_4 as stop solution was used to detect the response and optical densities read at 450 nm.

Tissue and cell recovery and isolation

At specified time points after immunization, blood samples were taken by tail vein bleed, and sera were isolated after clotting by centrifugation. Mice were culled using 100 μL intraperitoneal pentobarbitone (20-mg dose, Pentoject, Animalcare, UK), and tissues were collected as previously described.[15] Blood was collected from carotid vessels, and sera were isolated after clotting by centrifugation. Lungs were removed and homogenized by passage through 100-μm cell strainers, then centrifuged at 200 × g for 5 min. Supernatants were removed, and the cell pellet was treated with red blood cell lysis buffer (ACK; 0.15 M ammonium chloride, 1 M potassium hydrogen carbonate, and 0.01 mM EDTA, pH 7.2) before centrifugation at 200 × *g* for 5 min. The remaining cells were resuspended in RPMI 1640 medium with 10% fetal calf serum, and viable cell numbers were determined by trypan blue exclusion.

T cell ELISPOT

IFN-γ ELISPOT assays were performed using a commercial kit from AbCam (ab64029) following the manufacturer's recommendations. Cells were stimulated with 2 μg/mL anti-CD28 (Clone 37.51: BD) and 15-mer sequences with 11 amino acids overlap peptides from influenza A (H1N1) HA (Peptivator; Miltenyi Biotech). The spots were counted using the AID iSpot reader and EliSpot Reader software V 7.0.

HAI assay

All samples were analyzed by HAI assay using A/California/7/2009 (H1N1) virus strain as described.[41] Serum samples were pre-treated with Receptor Destroying Enzyme (RDE: Denka Seiken) for 18 h at 37°C before inactivating the enzyme at 56°C for 1 hour. RDE-treated serum was 2-fold serially diluted across the plate with PBS and incubated with pre-diluted 4 hemagglutinating units virus per well for 15 min at room temperature. 100 μL of 0.5% turkey erythrocytes diluted in PBS was then added to each well, and the plate was incubated for 30 min at room temperature before scoring the response.

Influenza viral load

Viral load *in vivo* was assessed by Trizol extraction of RNA from frozen lung tissue disrupted in a TissueLyzer (Qiagen, Manchester, UK). RNA was converted into cDNA, and quantitative RT-PCR was carried out using bulk viral RNA for the influenza M gene and mRNA using 0.1 μM forward primer (5′-AAGACAAGACCAATYCTGTCACCTCT-3′), 0.1 μM reverse primer (5′-TCTACGYTGCAGTCCYCGCT-3′) and 0.2 μM probe (5′-FAM-TYACGCTCACCGTGCCCAGTG-TAMRA-3′) on a Stratagene Mx3005p (Agilent technologies, Santa Clara, CA, USA).[15] M-specific RNA copy number was determined using an influenza M gene standard plasmid.

Statistical analysis

Calculations as described in figure legends were performed using Prism 9 (GraphPad Software, La Jolla, CA, USA).

DATA AVAILABILITY

Data are available on request from the authors.

SUPPLEMENTAL INFORMATION

Supplemental information can be found online at https://doi.org/10.1016/j.omtn.2022.11.024.

ACKNOWLEDGMENTS

The authors thank Kasia S and Catherine U for assistance in the *in vivo* studies; Jennifer O'Connor, Ben Pierce, Michelle Kiely, and Carolyn Newell for support in the grant administration; Clement Bouton and Zijun Lin for RNA manufacture; and Lesley Rawlinson for lab management. This project was funded by CEPI. C.J. was funded by BBSRC grant BB/V013831/1. The graphical abstract was created using BioRender. For the purpose of open access, the author has applied a Creative Commons Attribution (CC BY) license to any author-accepted manuscript version arising.

AUTHOR CONTRIBUTIONS

J.T.: conceptualization, writing – original draft, visualization, supervision, investigation; D.S.: investigation; Z.W.: investigation; K.F.: investigation; J.B.: investigation; A.B.: resources; P.M.: conceptualization, resources; R.C. and M.Y.: investigation; C.B.F.: resources; M.B. and Y.K.T.: resources; C.J.: conceptualization, resources, writing – reviewing & editing; R.S.: conceptualization, resources, writing – reviewing & editing.

DECLARATION OF INTERESTS

R.S., P.M., and A.B. are associated with Vaxequity global health and have stocks in it; M.B. and Y.T. are employed by Acuitas; C.F. was employed by IDRI.

REFERENCES

1. Tregoning, J.S., Flight, K.E., Higham, S.L., Wang, Z., and Pierce, B.F. (2021). Progress of the COVID-19 vaccine effort: viruses, vaccines and variants versus efficacy, effectiveness and escape. Nat. Rev. Immunol. *21*, 626–636. https://doi.org/10.1038/s41577-021-00592-1.
2. Tregoning, J.S., Brown, E.S., Cheeseman, H.M., Flight, K.E., Higham, S.L., Lemm, N.M., Pierce, B.F., Stirling, D.C., Wang, Z., and Pollock, K.M. (2020). Vaccines for COVID-19. Clin. Exp. Immunol. *202*, 162–192. https://doi.org/10.1111/cei.13517.
3. Vogel, A.B., Lambert, L., Kinnear, E., Busse, D., Erbar, S., Reuter, K.C., Wicke, L., Perkovic, M., Beissert, T., Haas, H., et al. (2018). Self-amplifying RNA vaccines give equivalent protection against influenza to mRNA vaccines but at much lower doses. Mol. Ther. *26*, 446–455. https://doi.org/10.1016/j.ymthe.2017.11.017.
4. McKay, P.F., Hu, K., Blakney, A.K., Samnuan, K., Brown, J.C., Penn, R., Zhou, J., Bouton, C.R., Rogers, P., Polra, K., et al. (2020). Self-amplifying RNA SARS-CoV-2 lipid nanoparticle vaccine candidate induces high neutralizing antibody titers in mice. Nat. Commun. *11*, 3523. https://doi.org/10.1038/s41467-020-17409-9.
5. Pollock, K.M., Cheeseman, H.M., Szubert, A.J., Libri, V., Boffito, M., Owen, D., Bern, H., O'Hara, J., McFarlane, L.R., Lemm, N.M., et al. (2022). Safety and immunogenicity of a self-amplifying RNA vaccine against COVID-19: COVAC1, a phase I, dose-ranging trial. EClinicalMedicine *44*, 101262. https://doi.org/10.1016/j.eclinm.2021.101262.
6. Maruggi G, Ulmer JB, Rappuoli R, Yu D. Self-amplifying mRNA-Based Vaccine Technology and its Mode of Action. Springer Berlin; 1-40.
7. Liu, G., and Gack, M.U. (2020). Distinct and orchestrated functions of RNA sensors in innate immunity. Immunity *53*, 26–42. https://doi.org/10.1016/S0140-6736(22)00791-7.
8. van Boxel-Dezaire, A.H.H., Rani, M.R.S., and Stark, G.R. (2006). Complex modulation of cell type-specific signaling in response to type I interferons. Immunity *25*, 361–372. https://doi.org/10.1016/j.immuni.2006.08.014.
9. Minnaert, A.-K., Vanluchene, H., Verbeke, R., Lentacker, I., De Smedt, S.C., Raemdonck, K., Sanders, N.N., and Remaut, K. (2021). Strategies for controlling the innate immune activity of conventional and self-amplifying mRNA therapeutics: getting the message across. Adv. Drug Deliv. Rev. *176*, 113900. https://doi.org/10.1016/j.addr.2021.113900.
10. McDonald, J.U., Zhong, Z., Groves, H.T., and Tregoning, J.S. (2017). Inflammatory responses to influenza vaccination at the extremes of age. Immunology *151*, 451–463. https://doi.org/10.1111/imm.12742.
11. Bloom, K., van den Berg, F., and Arbuthnot, P. (2021). Self-amplifying RNA vaccines for infectious diseases. Gene Ther. *28*, 117–129. https://doi.org/10.1038/s41434-020-00204-y.
12. Blakney, A.K., Zhu, Y., McKay, P.F., Bouton, C.R., Yeow, J., Tang, J., Hu, K., Samnuan, K., Grigsby, C.L., Shattock, R.J., and Stevens, M.M. (2020). Big is beautiful: enhanced saRNA delivery and immunogenicity by a higher molecular weight, bioreducible, cationic polymer. ACS Nano *14*, 5711–5727. https://doi.org/10.1021/acsnano.0c00326.
13. Erasmus, J.H., Khandhar, A.P., Guderian, J., Granger, B., Archer, J., Archer, M., Gage, E., Fuerte-Stone, J., Larson, E., Lin, S., et al. (2018). A nanostructured lipid carrier for delivery of a replicating viral RNA provides single, low-dose protection against Zika. Mol. Ther. *26*, 2507–2522. https://doi.org/10.1016/j.ymthe.2018.07.010.
14. Blakney, A.K., McKay, P.F., Hu, K., Samnuan, K., Jain, N., Brown, A., Thomas, A., Rogers, P., Polra, K., Sallah, H., et al. (2021). Polymeric and lipid nanoparticles for delivery of self-amplifying RNA vaccines. J. Control. Release *338*, 201–210. https://doi.org/10.1016/j.jconrel.2021.08.029.
15. Groves, H.T., McDonald, J.U., Langat, P., Kinnear, E., Kellam, P., McCauley, J., Ellis, J., Thompson, C., Elderfield, R., Parker, L., et al. (2018). Mouse models of influenza infection with circulating strains to test seasonal vaccine efficacy. Original research

frontiers in immunology. Front. Immunol. *9*, 126. https://doi.org/10.3389/fimmu.2018.00126.

16. Arimori, Y., Nakamura, R., Yamada, H., Shibata, K., Maeda, N., Kase, T., and Yoshikai, Y. (2013). Type I interferon limits influenza virus-induced acute lung injury by regulation of excessive inflammation in mice. Antiviral Res. *99*, 230–237. https://doi.org/10.1016/j.antiviral.2013.05.007.

17. Seo, S.-U., Kwon, H.-J., Song, J.-H., Byun, Y.-H., Seong, B.L., Kawai, T., Akira, S., and Kweon, M.-N. (2010). MyD88 signaling is indispensable for primary influenza A virus infection but dispensable for secondary infection. J. Virol. *84*, 12713–12722. https://doi.org/10.1128/JVI.01675-10.

18. Kandasamy, M., Suryawanshi, A., Tundup, S., Perez, J.T., Schmolke, M., Manicassamy, S., and Manicassamy, B. (2016). RIG-I signaling is critical for efficient polyfunctional T cell responses during influenza virus infection. PLoS Pathog. *12*, e100574. https://doi.org/10.1371/journal.ppat.1005754.

19. Lutz, J., Lazzaro, S., Habbeddine, M., Schmidt, K.E., Baumhof, P., Mui, B.L., Tam, Y.K., Madden, T.D., Hope, M.J., Heidenreich, R., and Fotin-Mleczek, M. (2017). Unmodified mRNA in LNPs constitutes a competitive technology for prophylactic vaccines. NPJ Vaccines *2*, 29. https://doi.org/10.1038/s41541-017-0032-6.

20. Liang, F., Lindgren, G., Lin, A., Thompson, E.A., Ols, S., Röhss, J., John, S., Hassett, K., Yuzhakov, O., Bahl, K., et al. (2017). Efficient targeting and activation of antigen-presenting cells in vivo after modified mRNA vaccine administration in rhesus macaques. Mol. Ther. *25*, 2635–2647. https://doi.org/10.1016/j.ymthe.2017.08.006.

21. Pepini, T., Pulichino, A.M., Carsillo, T., Carlson, A.L., Sari-Sarraf, F., Ramsauer, K., Debasitis, J.C., Maruggi, G., Otten, G.R., Geall, A.J., et al. (2017). Induction of an IFN-mediated antiviral response by a self-amplifying RNA vaccine: implications for vaccine design. J. Immunol. *198*, 4012–4024. https://doi.org/10.4049/jimmunol.1601877.

22. Ndeupen, S., Qin, Z., Jacobsen, S., Estanbouli, H., Bouteau, A., and Igyártó, B.Z. (2021). The mRNA-LNP platform's lipid nanoparticle component used in preclinical vaccine studies is highly inflammatory. Preprint at bioRxiv. https://doi.org/10.1101/2021.03.04.430128.

23. Shirai, S., Kawai, A., Shibuya, M., Munakata, L., Omata, D., Suzuki, R., and Yoshioka, Y. (2020). Lipid nanoparticle acts as a potential adjuvant for influenza split vaccine without inducing inflammatory responses. Vaccines *8*, 433. https://doi.org/10.3390/vaccines8030433.

24. Watson, D.S., Endsley, A.N., and Huang, L. (2012). Design considerations for liposomal vaccines: influence of formulation parameters on antibody and cell-mediated immune responses to liposome associated antigens. Vaccine *30*, 2256–2272. https://doi.org/10.1016/j.vaccine.2012.01.070.

25. Alameh, M.-G., Tombácz, I., Bettini, E., Lederer, K., Sittplangkoon, C., Wilmore, J.R., Gaudette, B.T., Soliman, O.Y., Pine, M., Hicks, P., et al. (2021). Lipid nanoparticles enhance the efficacy of mRNA and protein subunit vaccines by inducing robust T follicular helper cell and humoral responses. Immunity *54*, 2877–2892.e7. https://doi.org/10.1016/j.immuni.2021.11.001.

26. Huysmans, H., Zhong, Z., De Temmerman, J., Mui, B.L., Tam, Y.K., Mc Cafferty, S., Gitsels, A., Vanrompay, D., and Sanders, N.N. (2019). Expression kinetics and innate immune response after electroporation and LNP-mediated delivery of a self-amplifying mRNA in the skin. Mol. Ther. Nucleic Acids *17*, 867–878. https://doi.org/10.1016/j.omtn.2019.08.001.

27. Lazear, H.M., Lancaster, A., Wilkins, C., Suthar, M.S., Huang, A., Vick, S.C., Clepper, L., Thackray, L., Brassil, M.M., Virgin, H.W., et al. (2013). IRF-3, IRF-5, and IRF-7 coordinately regulate the type I IFN response in myeloid dendritic cells downstream of MAVS signaling. PLoS Pathog. *9*, e1003118. https://doi.org/10.1371/journal.ppat.1003118.

28. Zhang, H., You, X., Wang, X., Cui, L., Wang, Z., Xu, F., Li, M., Yang, Z., Liu, J., Huang, P., et al. (2021). Delivery of mRNA vaccine with a lipid-like material potentiates antitumor efficacy through Toll-like receptor 4 signaling. Proc. Natl. Acad. Sci. USA *118*. e2005191118. https://doi.org/10.1073/pnas.2005191118.

29. Blakney, A.K., McKay, P.F., Bouton, C.R., Hu, K., Samnuan, K., and Shattock, R.J. (2021). Innate inhibiting proteins enhance expression and immunogenicity of self-amplifying RNA. Mol. Ther. *29*, 1174–1185. https://doi.org/10.1016/j.ymthe.2020.11.011.

30. Bergamaschi, C., Terpos, E., Rosati, M., Angel, M., Bear, J., Stellas, D., Karaliota, S., Apostolakou, F., Bagratuni, T., Patseas, D., et al. (2021). Systemic IL-15, IFN-γ, and IP-10/CXCL10 signature associated with effective immune response to SARS-CoV-2 in BNT162b2 mRNA vaccine recipients. Cell Rep. *36*, 109504. https://doi.org/10.1016/j.celrep.2021.109504.

31. Fischetti, L., Zhong, Z., Pinder, C.L., Tregoning, J.S., and Shattock, R.J. (2017). The synergistic effects of combining TLR ligand based adjuvants on the cytokine response are dependent upon p38/JNK signalling. Cytokine *99*, 287–296. https://doi.org/10.1016/j.cyto.2017.08.009.

32. McKay, P.F., Cizmeci, D., Aldon, Y., Maertzdorf, J., Weiner, J., Kaufmann, S.H., Lewis, D.J., van den Berg, R.A., Del Giudice, G., and Shattock, R.J. (2019). Identification of potential biomarkers of vaccine inflammation in mice. Elife *8*, e46149. https://doi.org/10.7554/eLife.46149.

33. Veazey, R.S., Siddiqui, A., Klein, K., Buffa, V., Fischetti, L., Doyle-Meyers, L., King, D.F., Tregoning, J.S., and Shattock, R.J. (2015). Evaluation of mucosal adjuvants and immunization routes for the induction of systemic and mucosal humoral immune responses in macaques. Hum. Vaccin. Immunother. *11*, 2913–2922. https://doi.org/10.1080/21645515.2015.1070998.

34. Li, C., Lee, A., Grigoryan, L., Arunachalam, P.S., Scott, M.K.D., Trisal, M., Wimmers, F., Sanyal, M., Weidenbacher, P.A., Feng, Y., et al. (2022). Mechanisms of innate and adaptive immunity to the Pfizer-BioNTech BNT162b2 vaccine. Nat. Immunol. *23*, 543–555. https://doi.org/10.1038/s41590-022-01163-9.

35. Davies, N., Hovdal, D., Edmunds, N., Nordberg, P., Dahlén, A., Dabkowska, A., Arteta, M.Y., Radulescu, A., Kjellman, T., Höijer, A., et al. (2021). Functionalized lipid nanoparticles for subcutaneous administration of mRNA to achieve systemic exposures of a therapeutic protein. Mol. Ther. Nucleic Acids *24*, 369–384. https://doi.org/10.1016/j.omtn.2021.03.008.

36. Barrat, F.J. (2018). TLR8: No gain, no pain. J. Exp. Med. *215*, 2964–2966. https://doi.org/10.1084/jem.20181899.

37. Liu, J., Xu, C., Hsu, L.-C., Luo, Y., Xiang, R., and Chuang, T.-H. (2010). A five-amino-acid motif in the undefined region of the TLR8 ectodomain is required for species-specific ligand recognition. Mol. Immunol. *47*, 1083–1090. https://doi.org/10.1016/j.molimm.2009.11.003.

38. McKay, P.F., Zhou, J., Frise, R., Blakney, A.K., Bouton, C.R., Wang, Z., Hu, K., Samnuan, K., Brown, J.C., Kugathasan, R., et al. (2022). Polymer formulated self-amplifying RNA vaccine is partially protective against influenza virus infection in ferrets. Oxford Open Immunol. 3, iqac004.

39. Goritzka, M., Durant, L.R., Pereira, C., Salek-Ardakani, S., Openshaw, P.J.M., and Johansson, C. (2014). Alpha/beta interferon receptor signaling amplifies early proinflammatory cytokine production in the lung during respiratory syncytial virus infection. J. Virol. *88*, 6128–6136. https://doi.org/10.1128/JVI.00333-14.

40. Kumagai, Y., Takeuchi, O., Kato, H., Kumar, H., Matsui, K., Morii, E., Aozasa, K., Kawai, T., and Akira, S. (2007). Alveolar macrophages are the primary interferon-alpha producer in pulmonary infection with RNA viruses. Immunity *27*, 240–252. https://doi.org/10.1016/j.immuni.2007.07.013.

41. Zhong, Z., Haltalli, M., Holder, B., Rice, T., Donaldson, B., O'Driscoll, M., Le-Doare, K., Kampmann, B., and Tregoning, J.S. (2019). The impact of timing of maternal influenza immunization on infant antibody levels at birth. Clin. Exp. Immunol. *195*, 139–152. https://doi.org/10.1111/cei.13234.

OMTN, Volume *31*

Supplemental information

Formulation, inflammation, and RNA sensing impact the immunogenicity of self-amplifying RNA vaccines

John S. Tregoning, David C. Stirling, Ziyin Wang, Katie E. Flight, Jonathan C. Brown, Anna K. Blakney, Paul F. McKay, Robert F. Cunliffe, Valarmathy Murugaiah, Christopher B. Fox, Mitchell Beattie, Ying K. Tam, Cecilia Johansson, and Robin J. Shattock

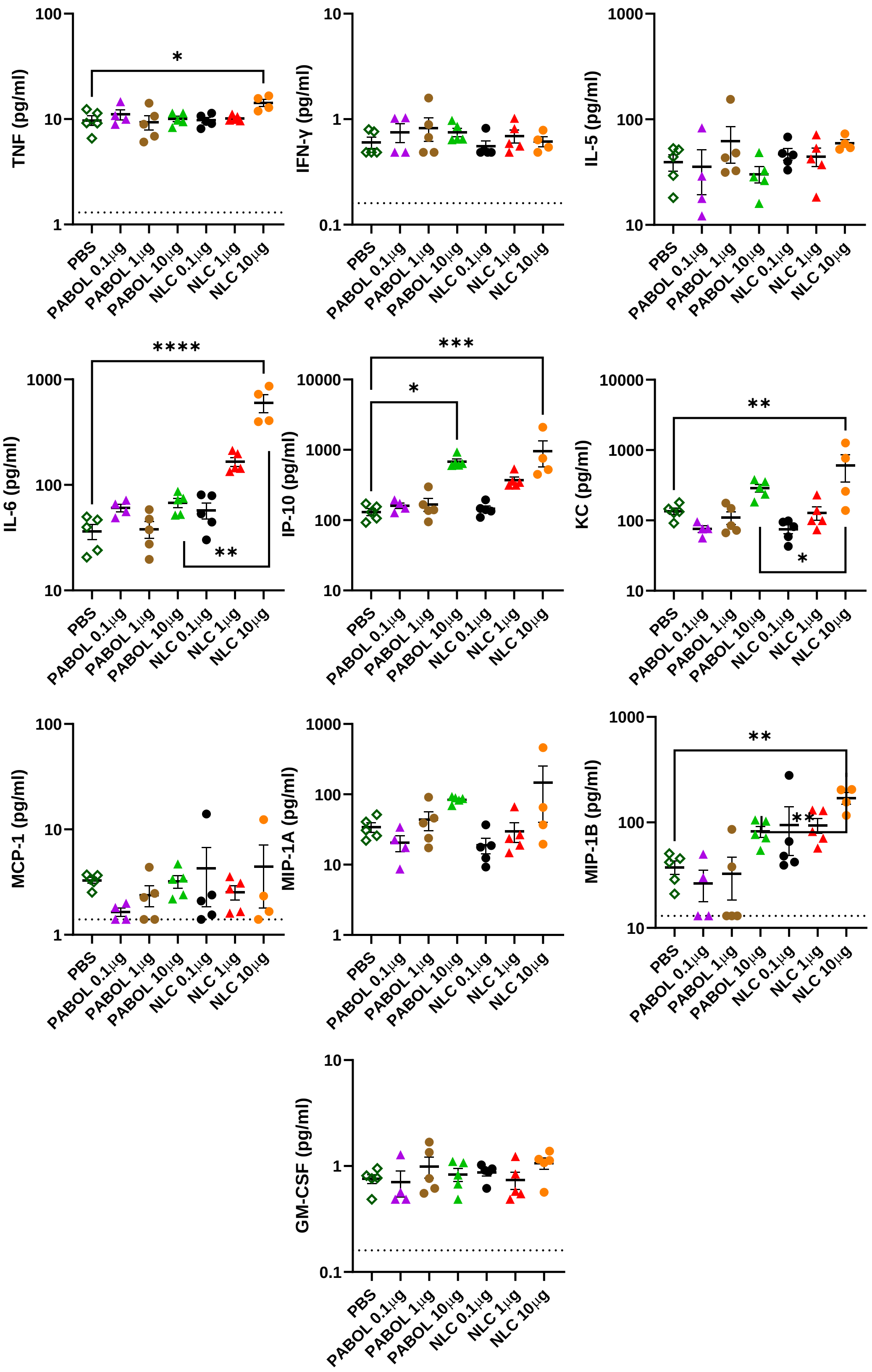

Figure S1. Comparison of blood cytokines after prime immunisation with NLC or pABOL formulated saRNA. Female C57BL/6 mice were intramuscularly immunised with increasing doses of saRNA encoding HA formulated with pABOL or in a nanostructured lipid carrier (NLC) at 0 and 4 weeks. Cytokines in blood were measured by MSD multiplex 4 hours after primary immunisation. Individual cytokine levels. Dotted line where present indicates limit of detection.

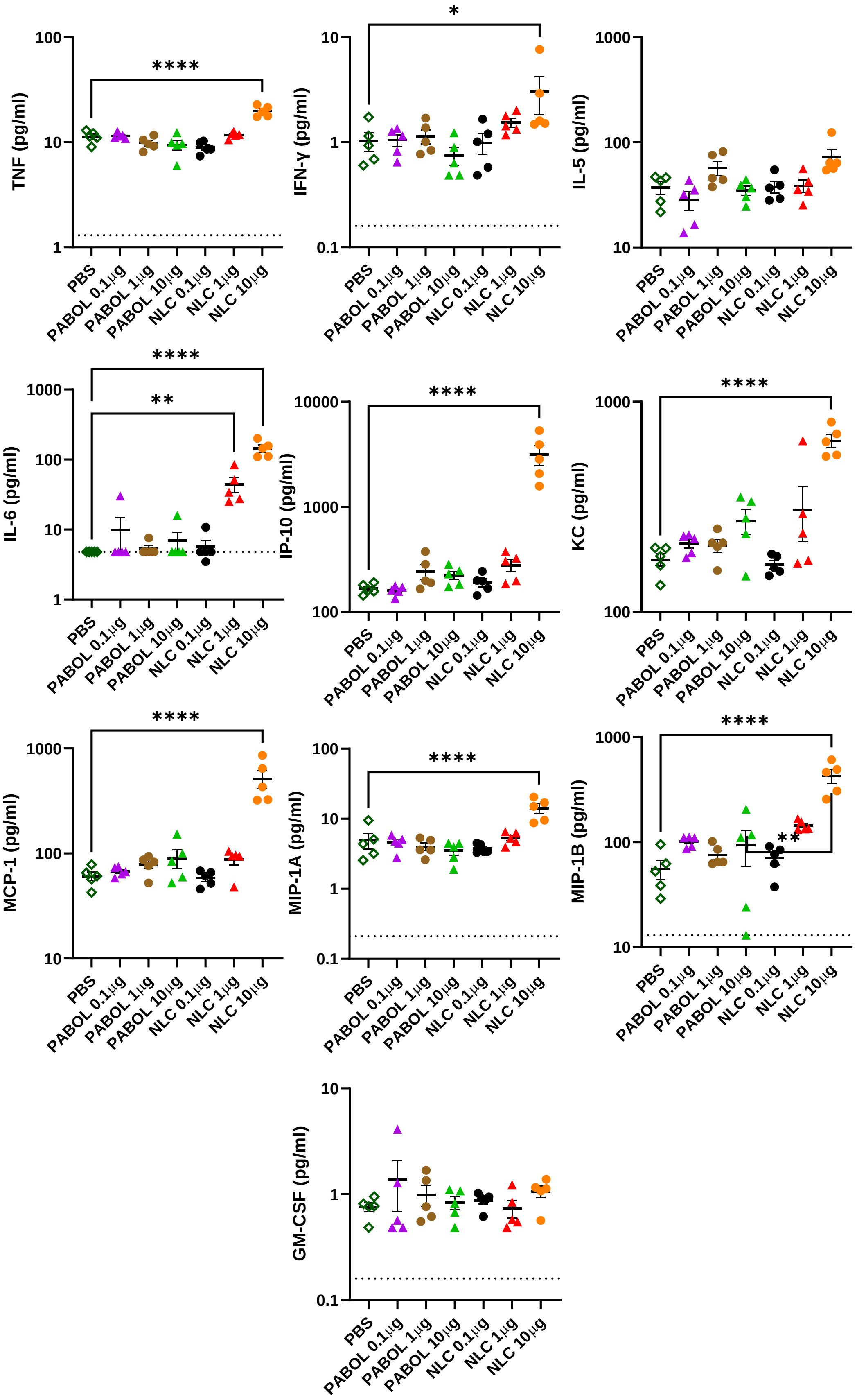

Figure S2. Comparison of blood cytokines after boost immunisation with NLC or pABOL formulated saRNA. Female C57BL/6 mice were intramuscularly immunised with increasing doses of saRNA encoding HA formulated with pABOL or in a nanostructured lipid carrier (NLC) at 0 and 4 weeks. Cytokines in blood were measured by MSD multiplex 4 hours after primary immunisation. Individual cytokine levels.

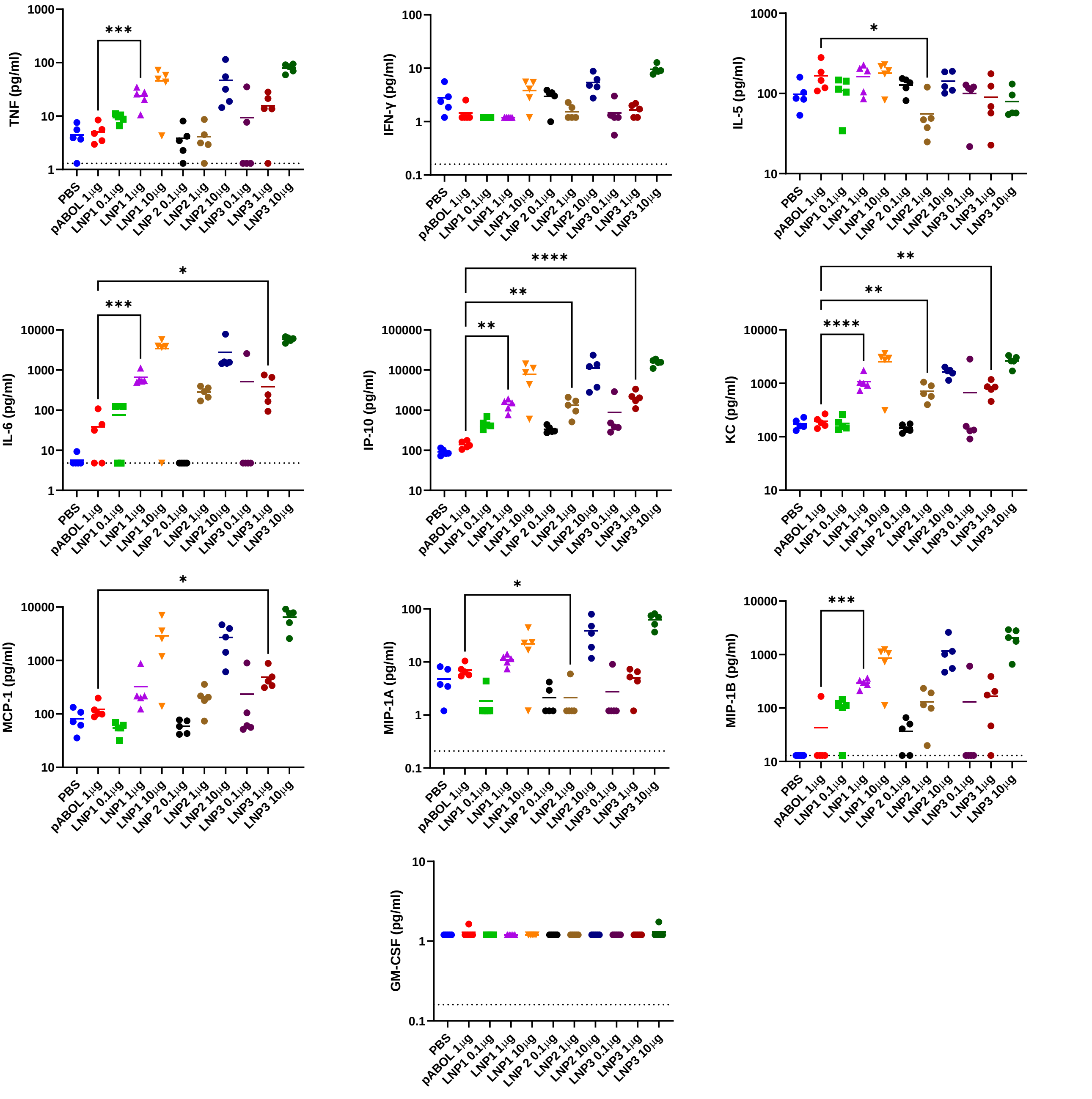

Figure S3. Comparison of blood cytokines after immunisation with LNP or pABOL formulated saRNA. Female C57BL/6 mice were intramuscularly immunised with increasing doses of saRNA encoding HA formulated with lipid nanoparticles (LNP) at 0 and 4 weeks. Cytokines in blood were measured by MSD multiplex 4 hours after primary immunisation. Individual cytokine levels.

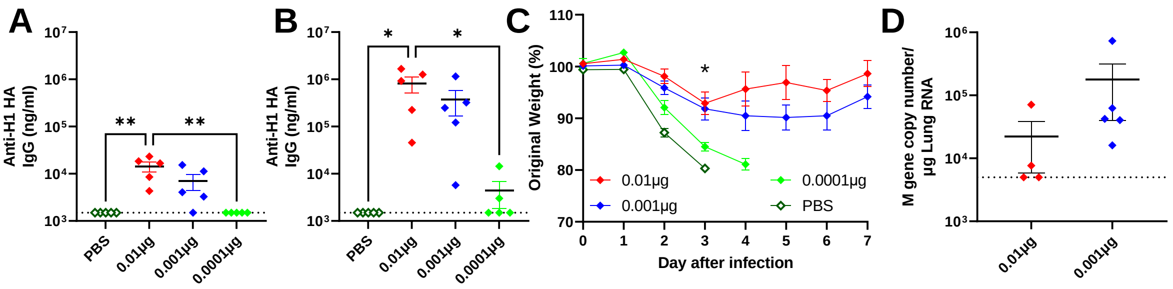

Figure S4. LNP formulated saRNA are protective at a very low dose. Female C57BL/6 mice were intramuscularly immunised with small doses of saRNA encoding HA formulated with lipid nanoparticles (LNP) at 0 and 4 weeks. Blood was collected to measure anti-HA antibody responses at 4 (A) and 6 weeks (B). Mice were infected intranasally with influenza virus at 6 weeks, weight loss was measured after infection (C). Viral load on d7; 0.0001µg and PBS immunised groups culled earlier (D). N=5 mice per group, points represent individual animals (A, B) or means (C), *p<0.05, ** p<0.01. Statistical analysis was performed by ANOVA with a Tukey test.

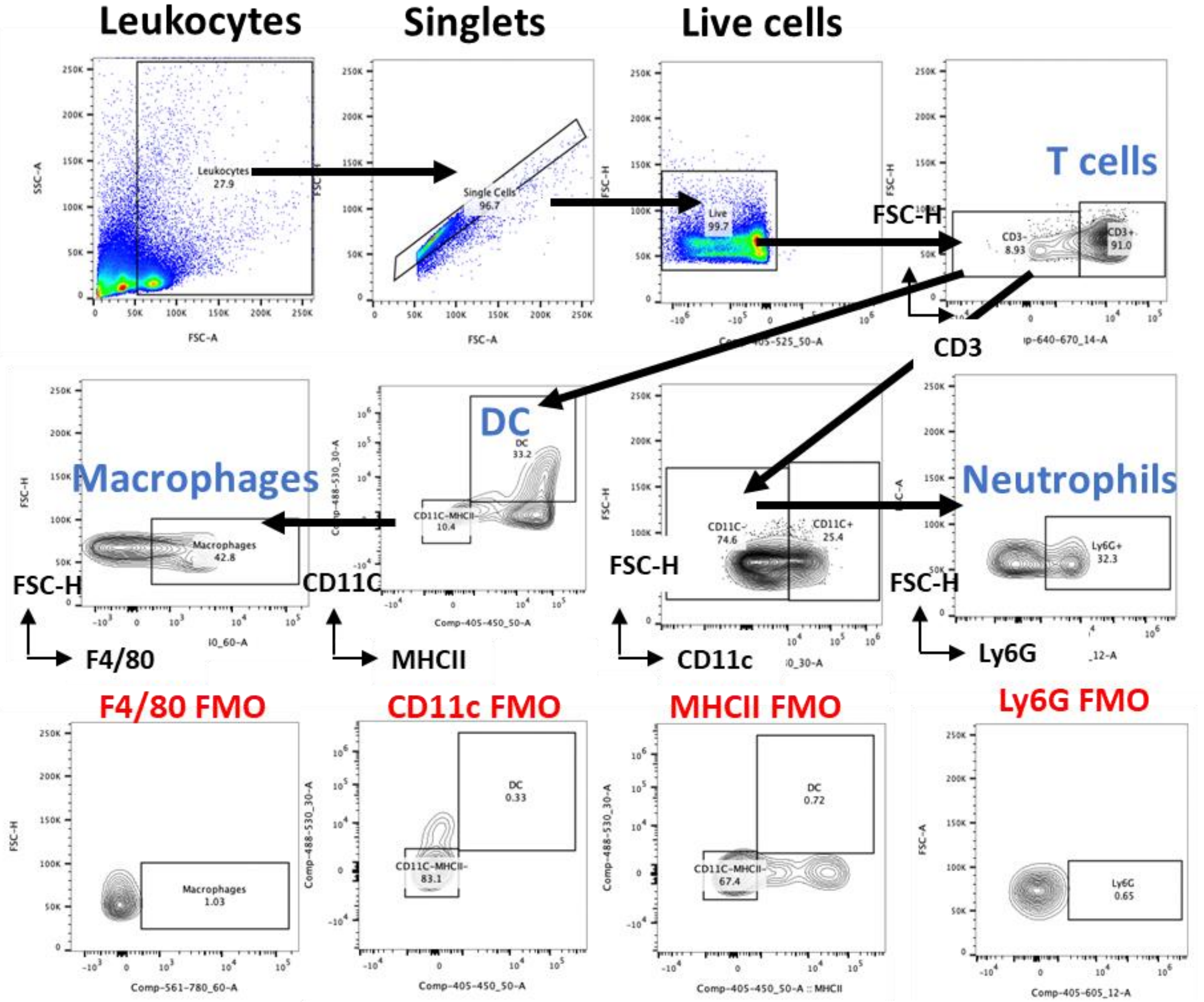

Figure S5. Gating strategy. Cells were extracted from lymph nodes prior to running by flow cytometry. Gating strategy for different cell types shown.

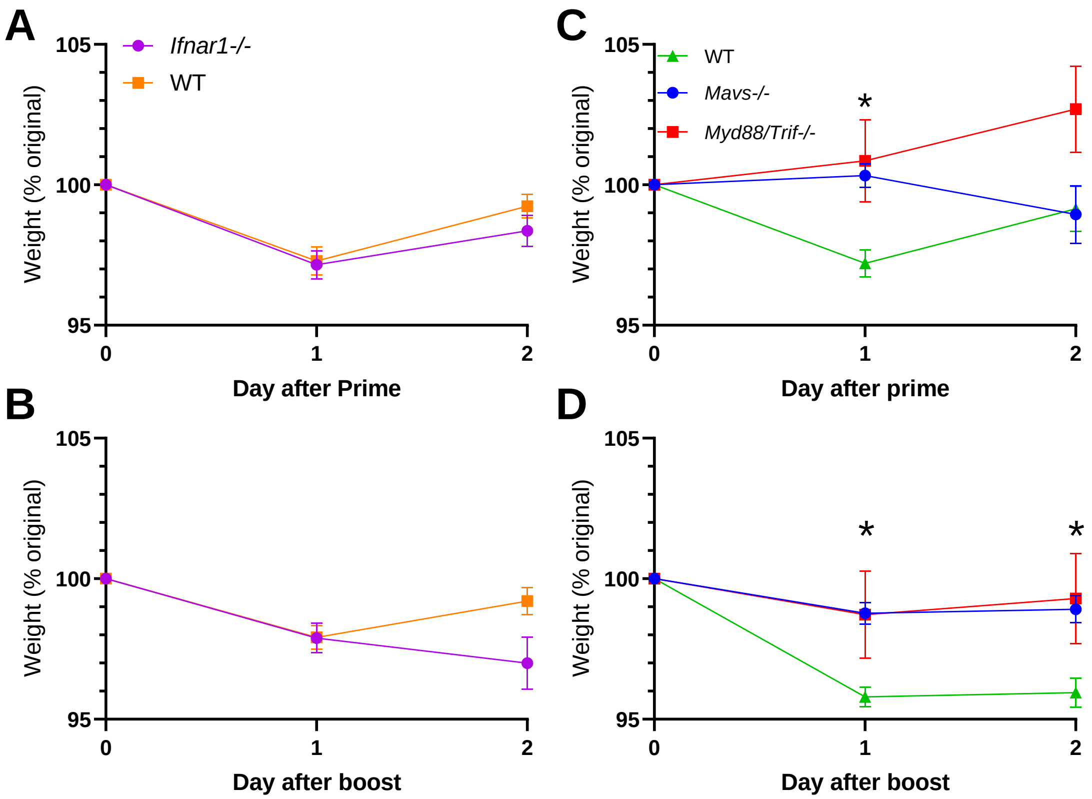

Figure S6. MAVS deficiency affects acute weight loss after vaccination. Wild type and *Ifnar*$^{-/-}$ mice were immunised with 1 µg saRNA formulated in LNP. Weight change was measured after prime (A) or boost immunisation (B). Wild type, *Mavs*$^{-/-}$ and *Myd88/Trif*$^{-/-}$ mice were immunised with 1 µg saRNA formulated in LNP. Weight change was measured after prime (C) or boost immunisation (D). *$p<0.05$ comparing *Mavs*$^{-/-}$ and wild type.
